# Supplementary material for: The zinc transporter Slc30a1 (ZnT1) in macrophages plays a protective role against attenuated Salmonella
Source: eLife. 2024 Oct 30;13:e89509. doi: 10.7554/eLife.89509 (PMC11524588; doi:10.7554/eLife.89509)
Supplement: Figure 2—source data 1. [file elife-89509-fig2-data1.zip › Figure 2-Source data 1/Figure 2-Source data 1_Raw images of western blot analysis for Slc30a1-flag expression.pdf]

Figure 2–Source data 1
Raw images of western blot analysis for Slc30a1-flag expression.

Western blot analysis of Slc30a1-flag in BMDMs isolated from *Slc30a1*$^{flag\text{-}EGFP/+}$mice for Figure 2B

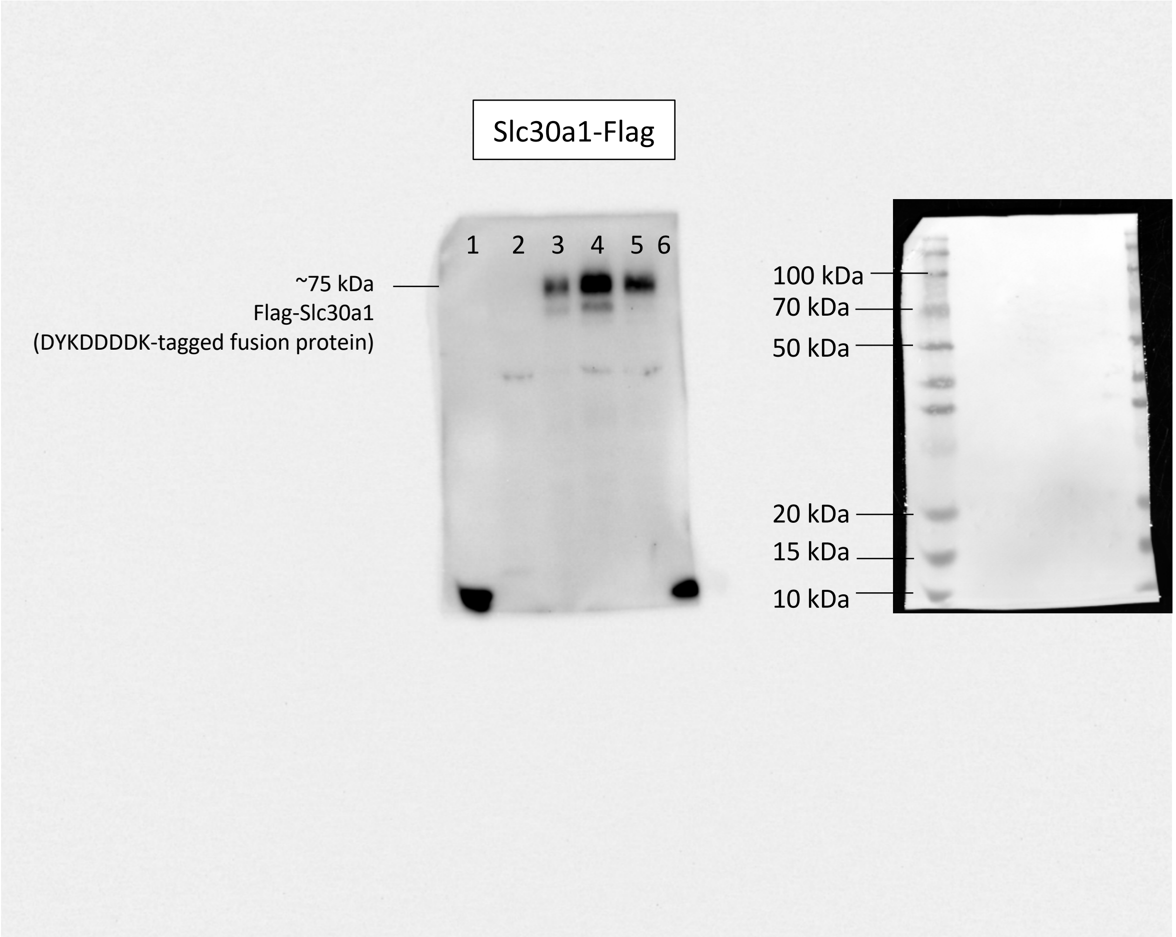

Lane 1: Ladder
Lane 2: Untreated (BMDM wild type)
Lane 3: Untreated
Lane 4: $ZnSO_4$ treatment (40 µM)
Lane 5: *Salmonella* (MOI = 1)
Lane 6: Ladder

Western blot analysis of β-Actin in BMDMs isolated from *Slc30a1*$^{flag\text{-}EGFP/+}$mice

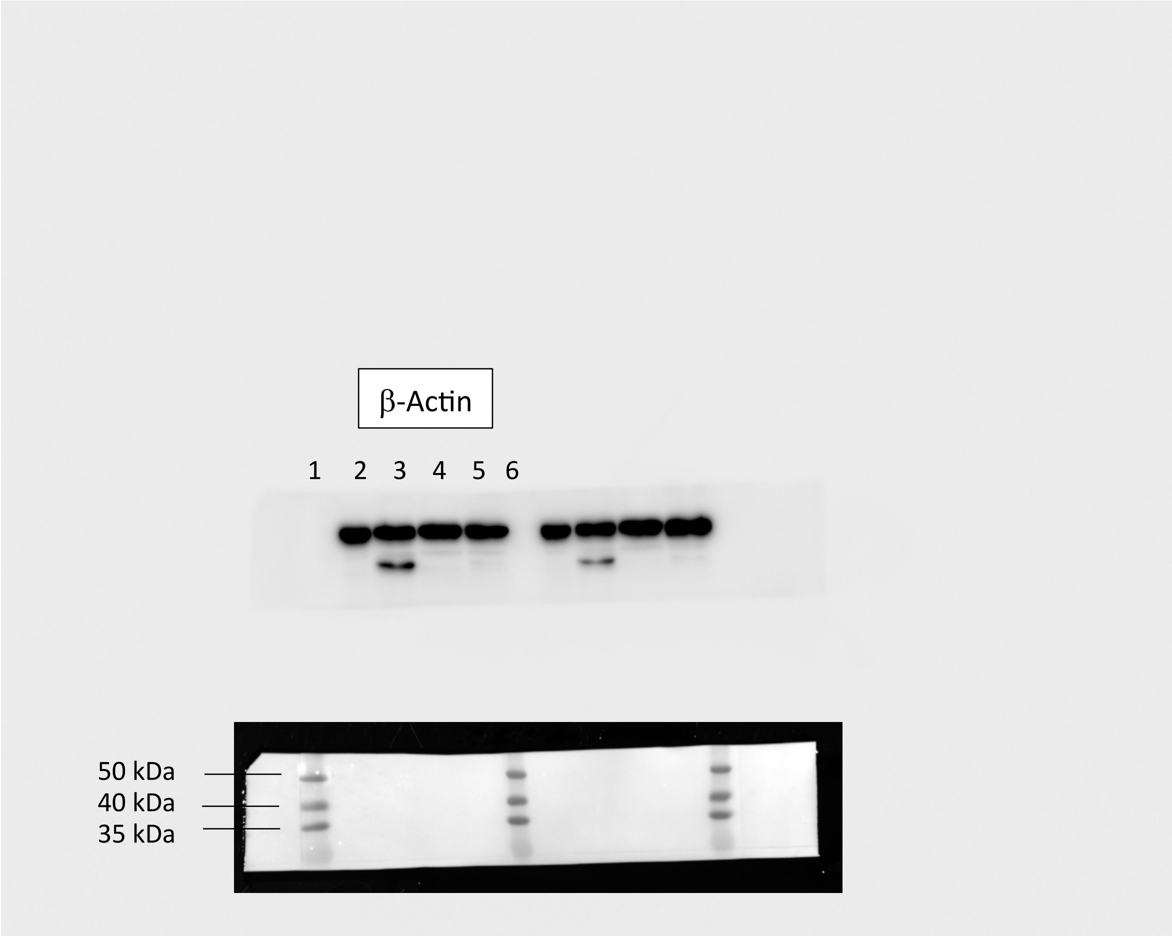

Lane 1: Ladder
Lane 2: Untreated (wild type)
Lane 3: Untreated
Lane 4: $ZnSO_4$ treatment (40 μM)
Lane 5: *Salmonella* (MOI = 1)
Lane 6: Ladder
