# Supplementary figures and images for: The zinc transporter Slc30a1 (ZnT1) in macrophages plays a protective role against attenuated Salmonella
Source: eLife. 2024 Oct 30;13:e89509. doi: 10.7554/eLife.89509 (PMC11524588; doi:10.7554/eLife.89509)

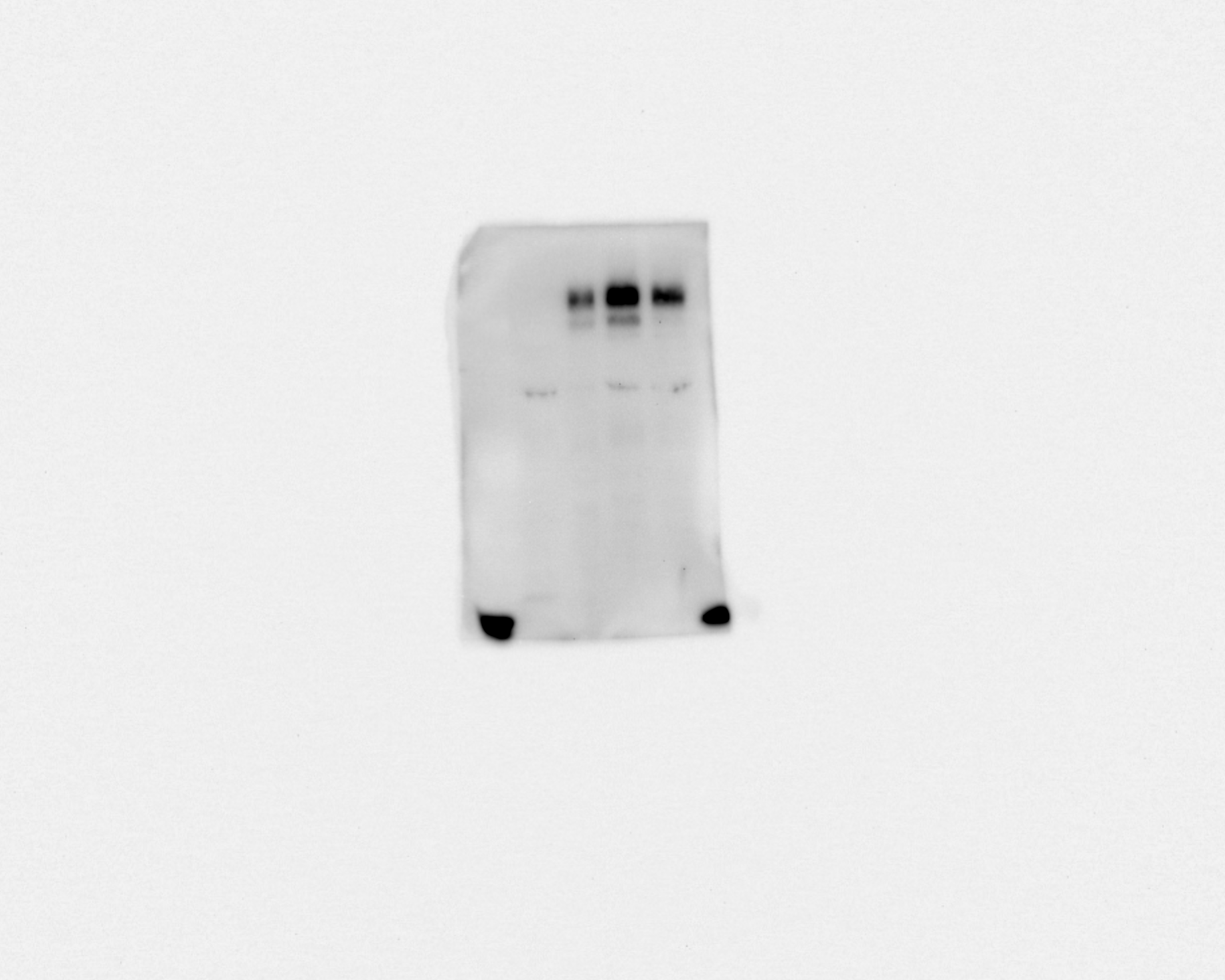

Supplement: Figure 2—source data 1. [file elife-89509-fig2-data1.zip › Figure 2-Source data 1/Figure 2-Source data 1-Unedited Blots-Slc30a1-Flag.tif]

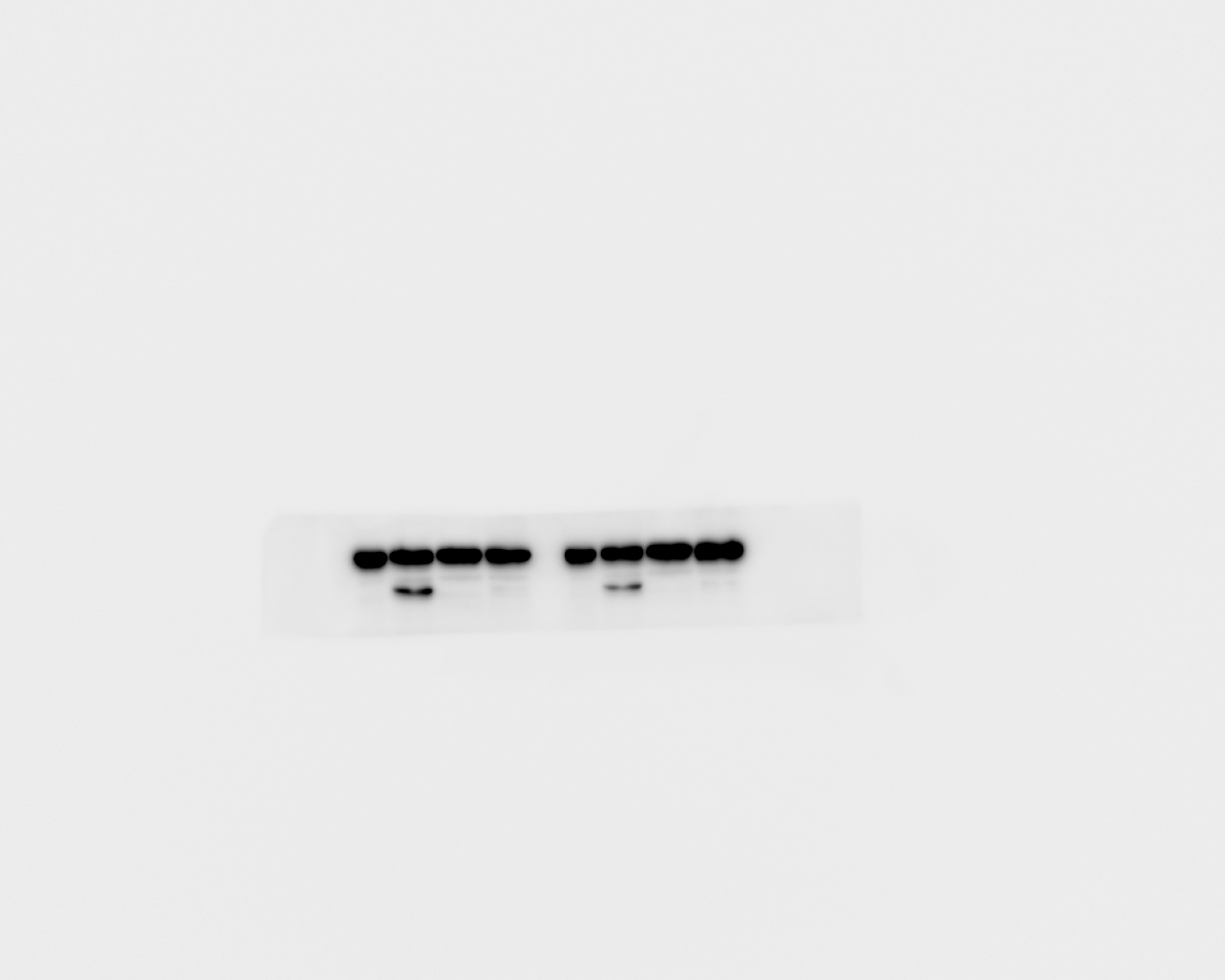

Supplement: Figure 2—source data 1. [file elife-89509-fig2-data1.zip › Figure 2-Source data 1/Figure 2-Source data 1-Unedited Blots-Beta-Actin .tif]

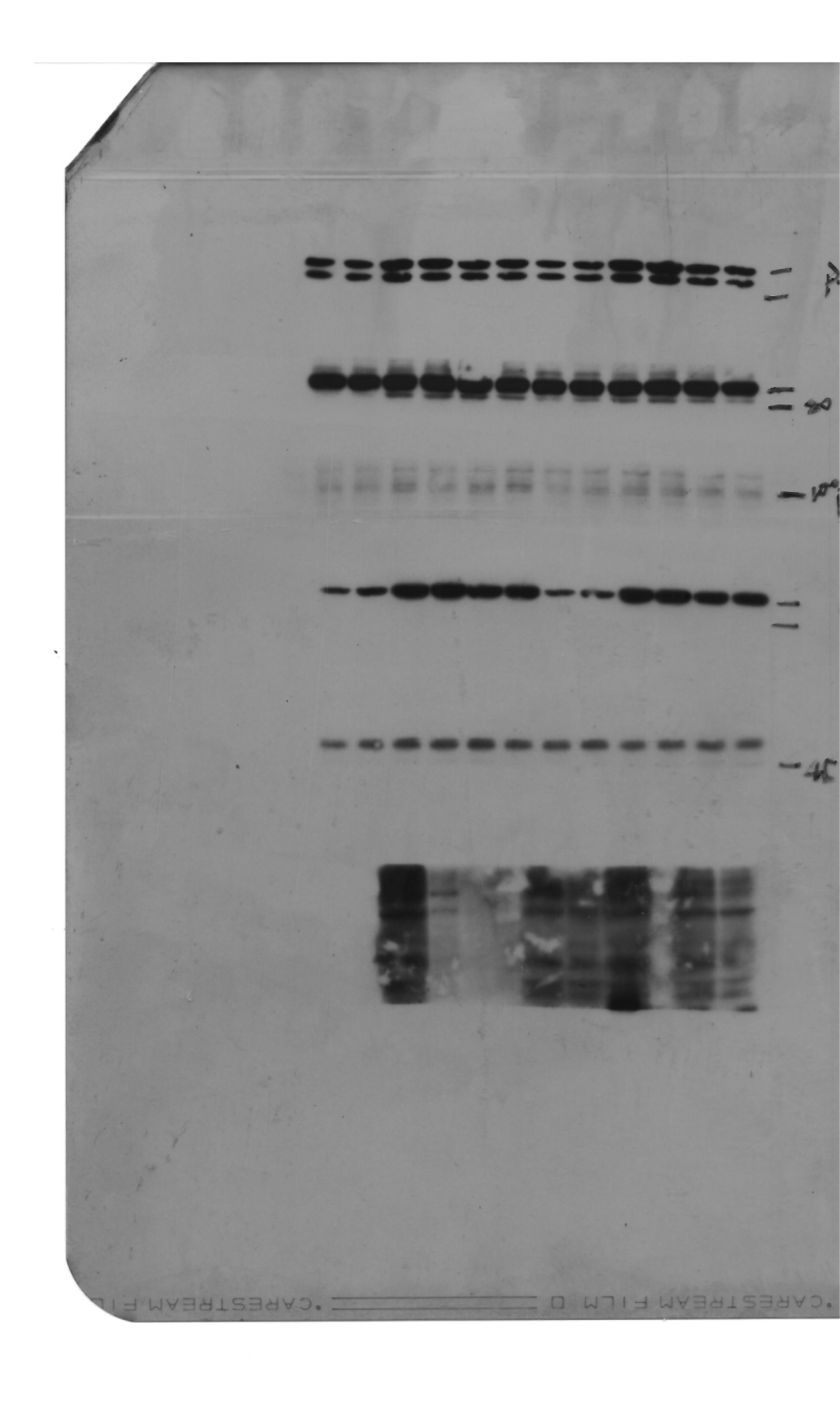

Supplement: Figure 5—source data 1. [file elife-89509-fig5-data1.zip › Figure 5-Figure supplement 1-Source data 2/Figure 5-Source data 2-for figure supplement 1F-Unedited Blots-NfKb-3(LPS).tiff]

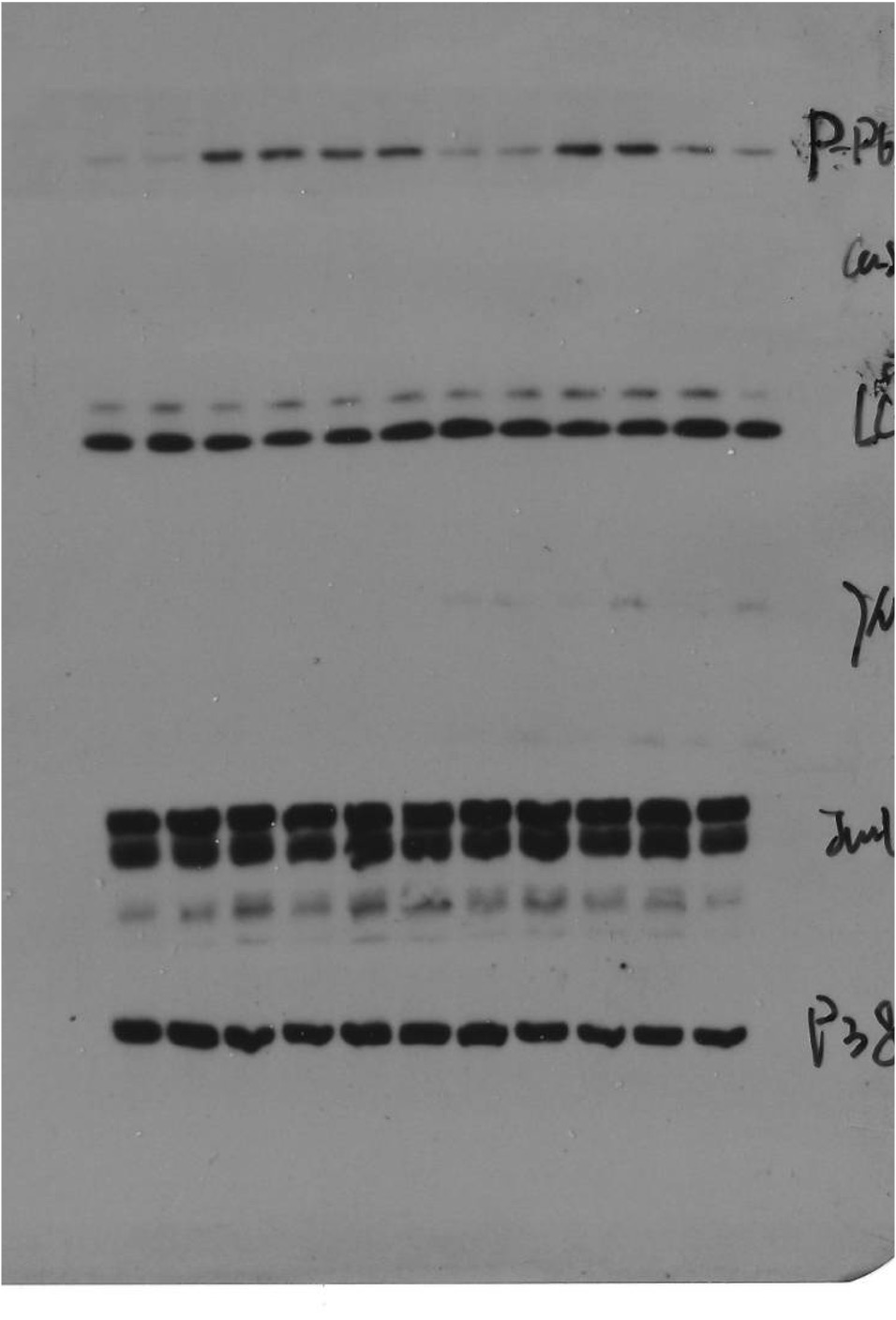

Supplement: Figure 5—source data 1. [file elife-89509-fig5-data1.zip › Figure 5-Figure supplement 1-Source data 2/Figure 5-Source data 2-for figure supplement 1F-Unedited Blots-Nfkb-2 (LPS).tiff]

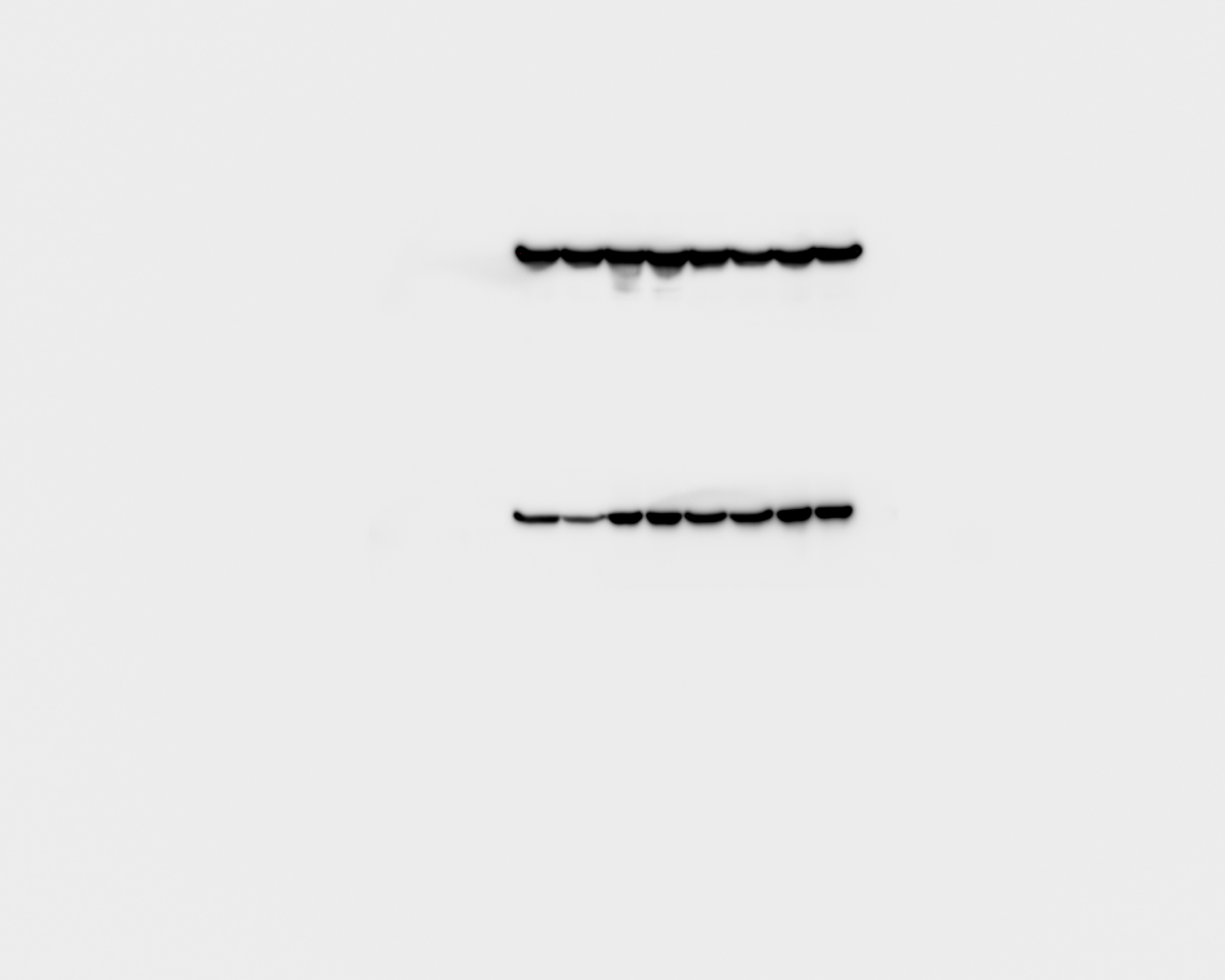

Supplement: Figure 5—source data 1. [file elife-89509-fig5-data1.zip › Figure 5-Figure supplement 1-Source data 2/Figure 5-Source data 2-for figure supplement 1D-Unedited Blots-Actin (LPS).tif]

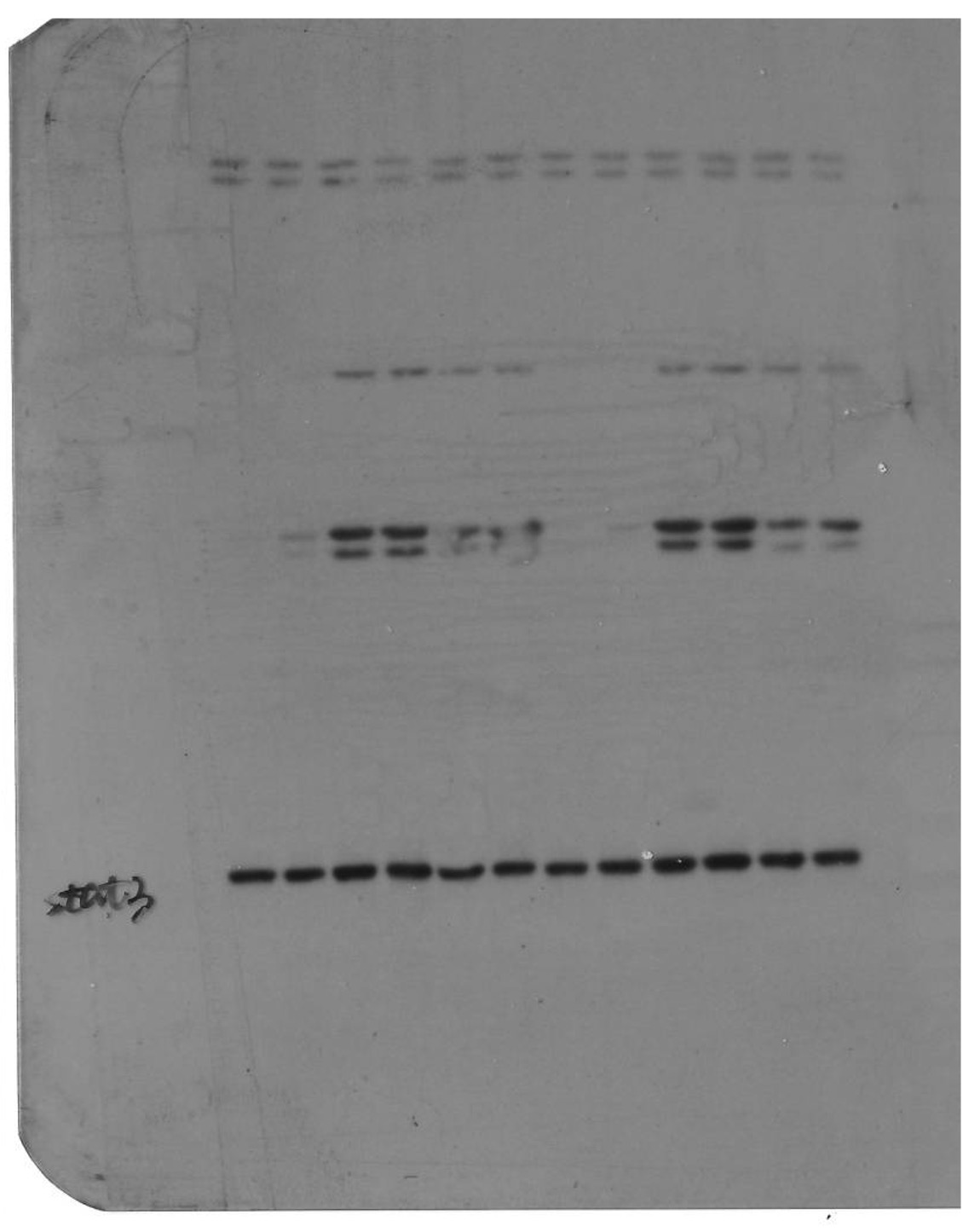

Supplement: Figure 5—source data 1. [file elife-89509-fig5-data1.zip › Figure 5-Figure supplement 1-Source data 2/Figure 5-Source data 2-for figure supplement 1F-Unedited Blots-Nfkb-1 (LPS).tiff]

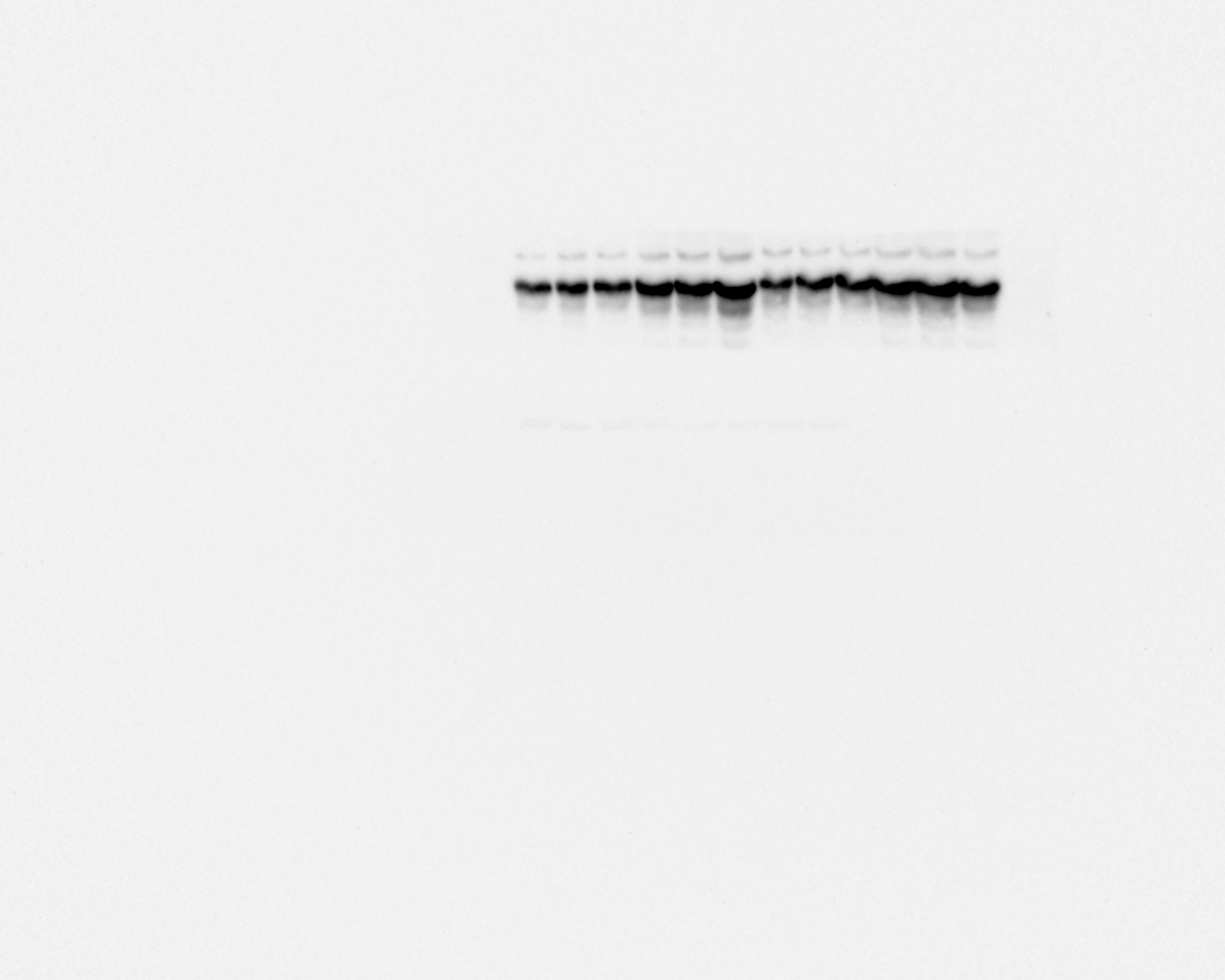

Supplement: Figure 5—source data 1. [file elife-89509-fig5-data1.zip › Figure 5-Figure supplement 1-Source data 2/Figure 5-Source data 2-for figure supplement 1C-Unedited Blots-Actin (HK-ST).tif]

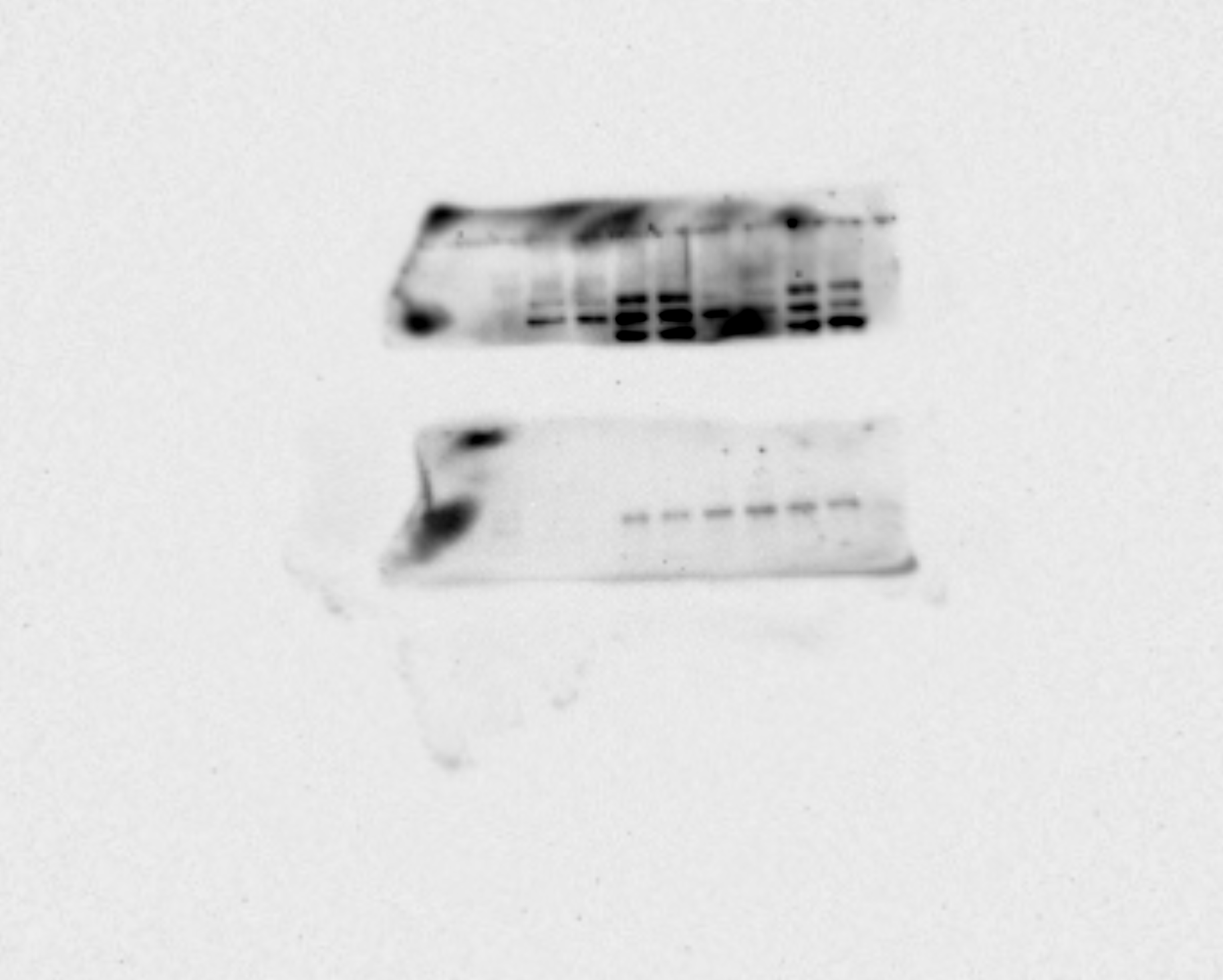

Supplement: Figure 5—source data 1. [file elife-89509-fig5-data1.zip › Figure 5-Figure supplement 1-Source data 2/Figure 5-Source data 2-for figure supplement 1D-Unedited Blots-iNOS (LPS).tif]

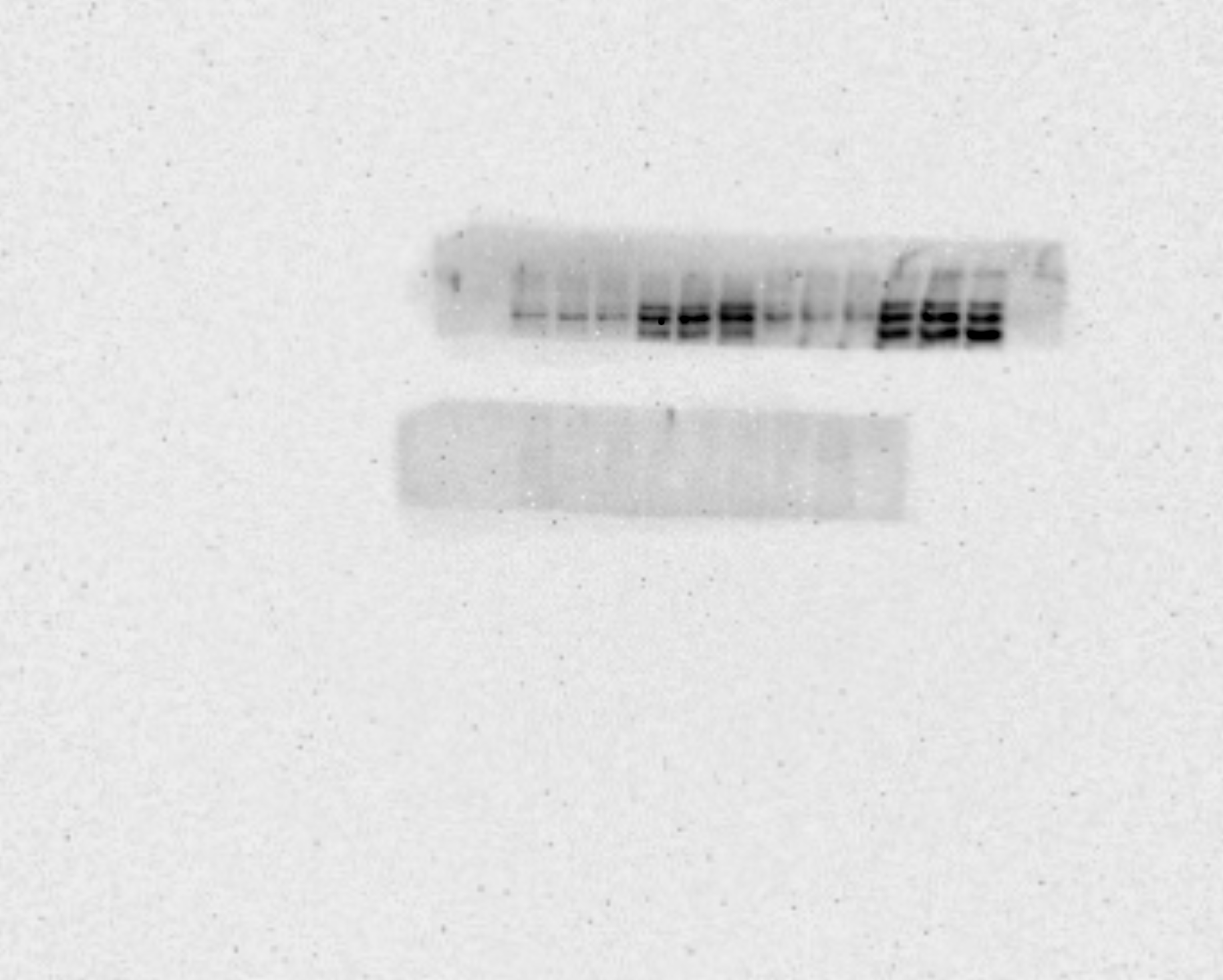

Supplement: Figure 5—source data 1. [file elife-89509-fig5-data1.zip › Figure 5-Figure supplement 1-Source data 2/Figure 5-Source data 2-for figure supplement 1C-Unedited Blots-iNOS (HK-ST).tif]

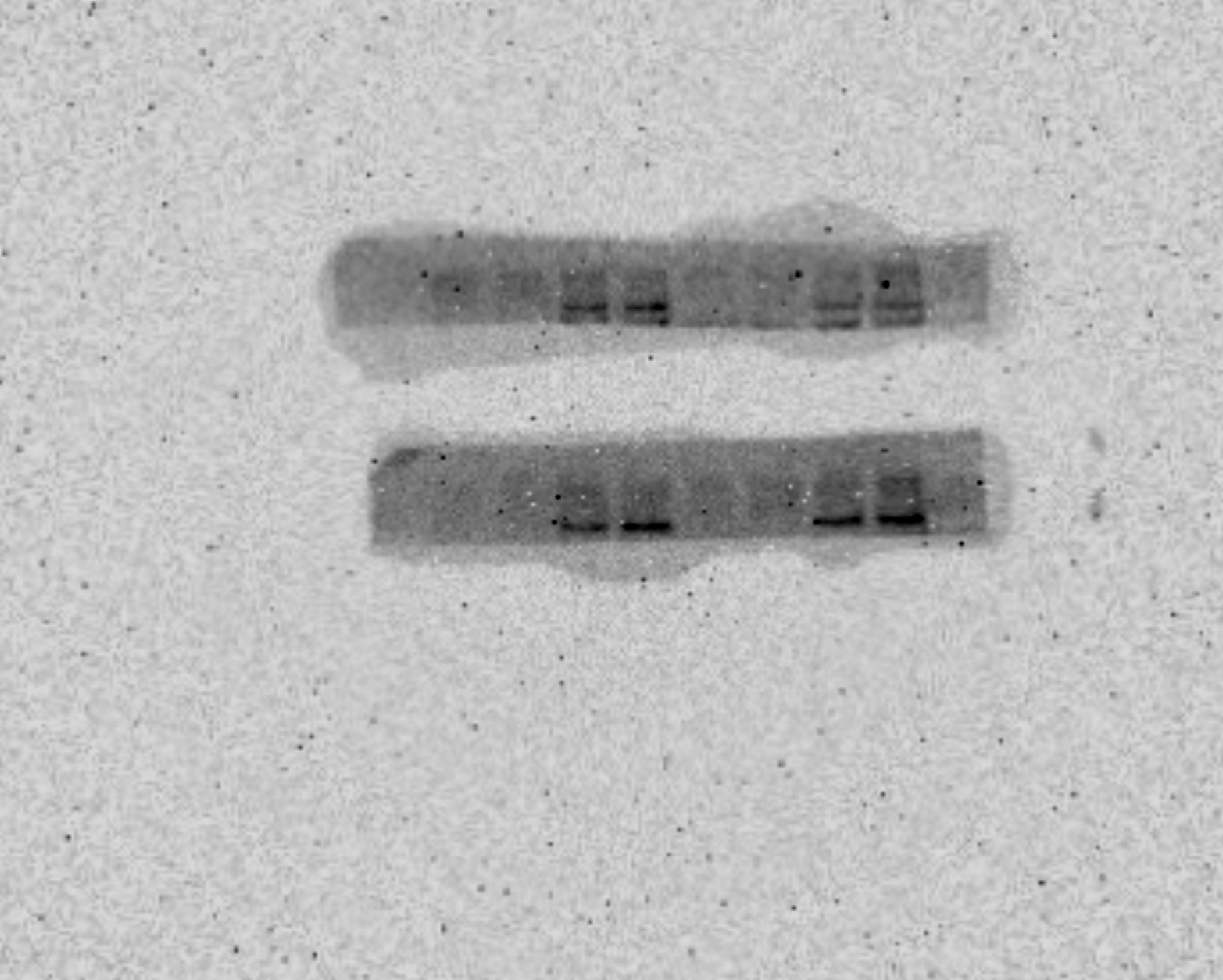

Supplement: Figure 5—source data 2. [file elife-89509-fig5-data2.zip › Figure 5-Source data 1/Figure 5-Source data 1-For Figure 5F-Unedited Blots-iNOS (ST).tif]

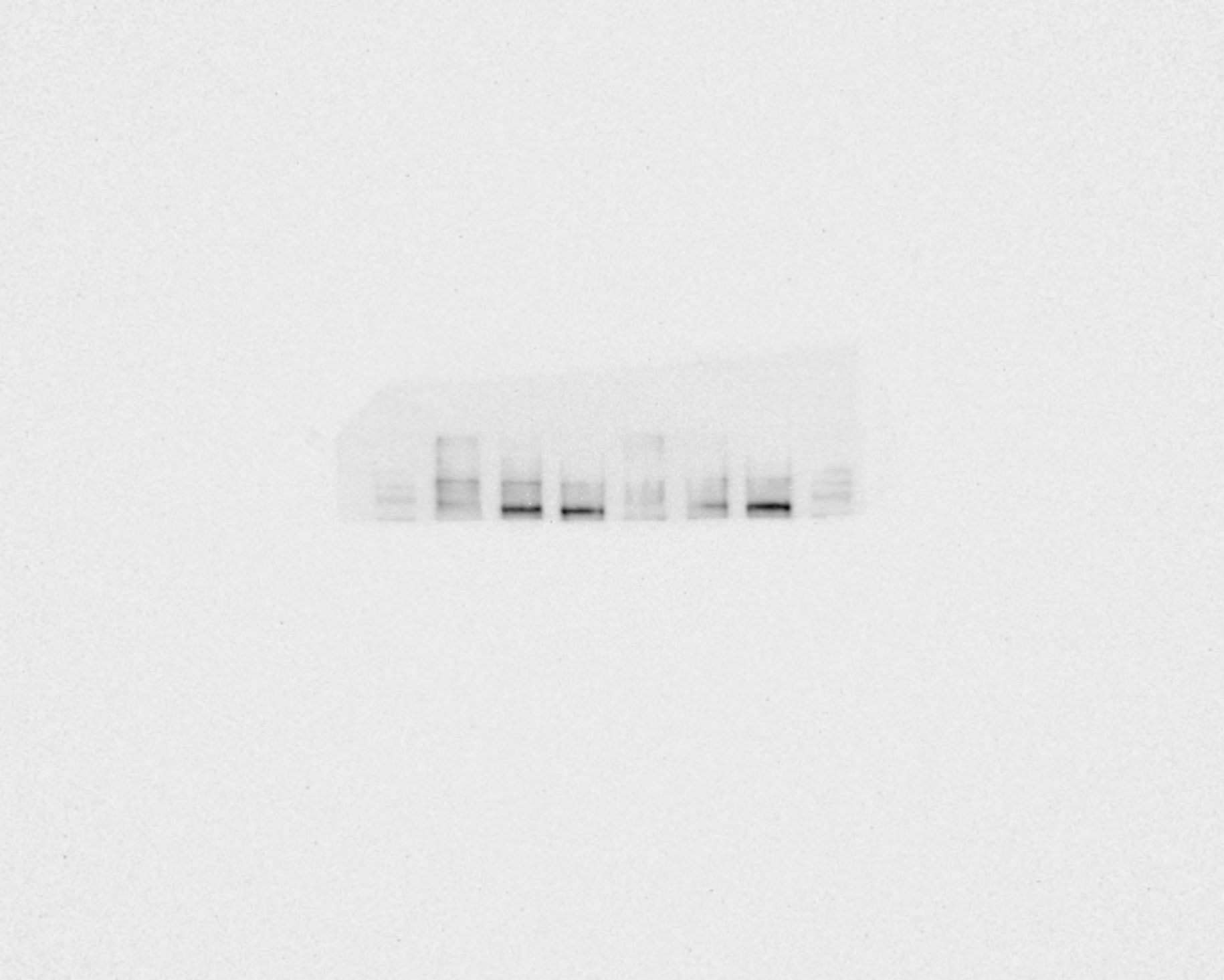

Supplement: Figure 5—source data 2. [file elife-89509-fig5-data2.zip › Figure 5-Source data 1/Figure 5-Source data 1 for Figure 5H-Unedited Blots-pp65 (ST).tif]

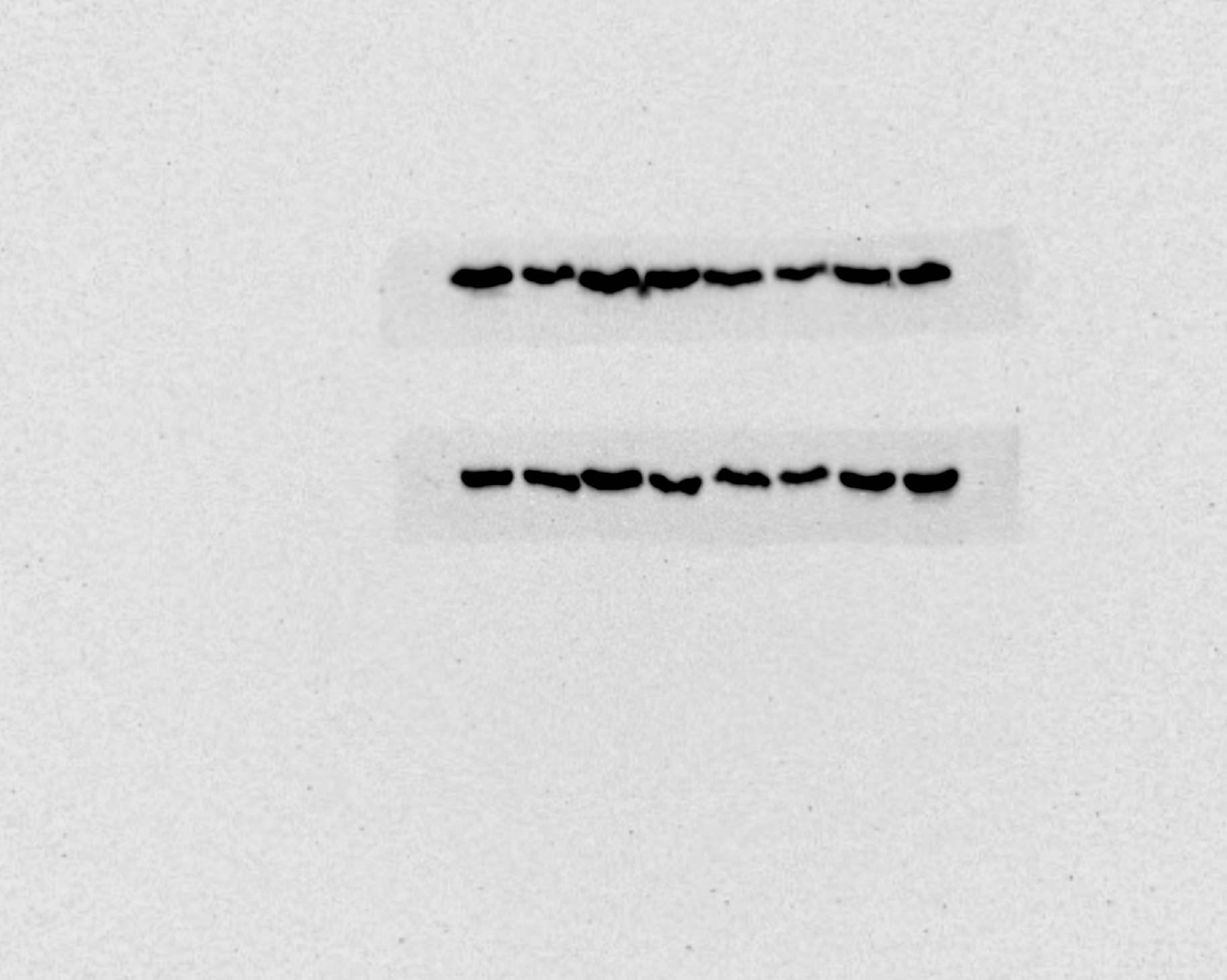

Supplement: Figure 5—source data 2. [file elife-89509-fig5-data2.zip › Figure 5-Source data 1/Figure 5-Source data 1-for Figure 5F-Unedited Blots-Actin (ST).tif]

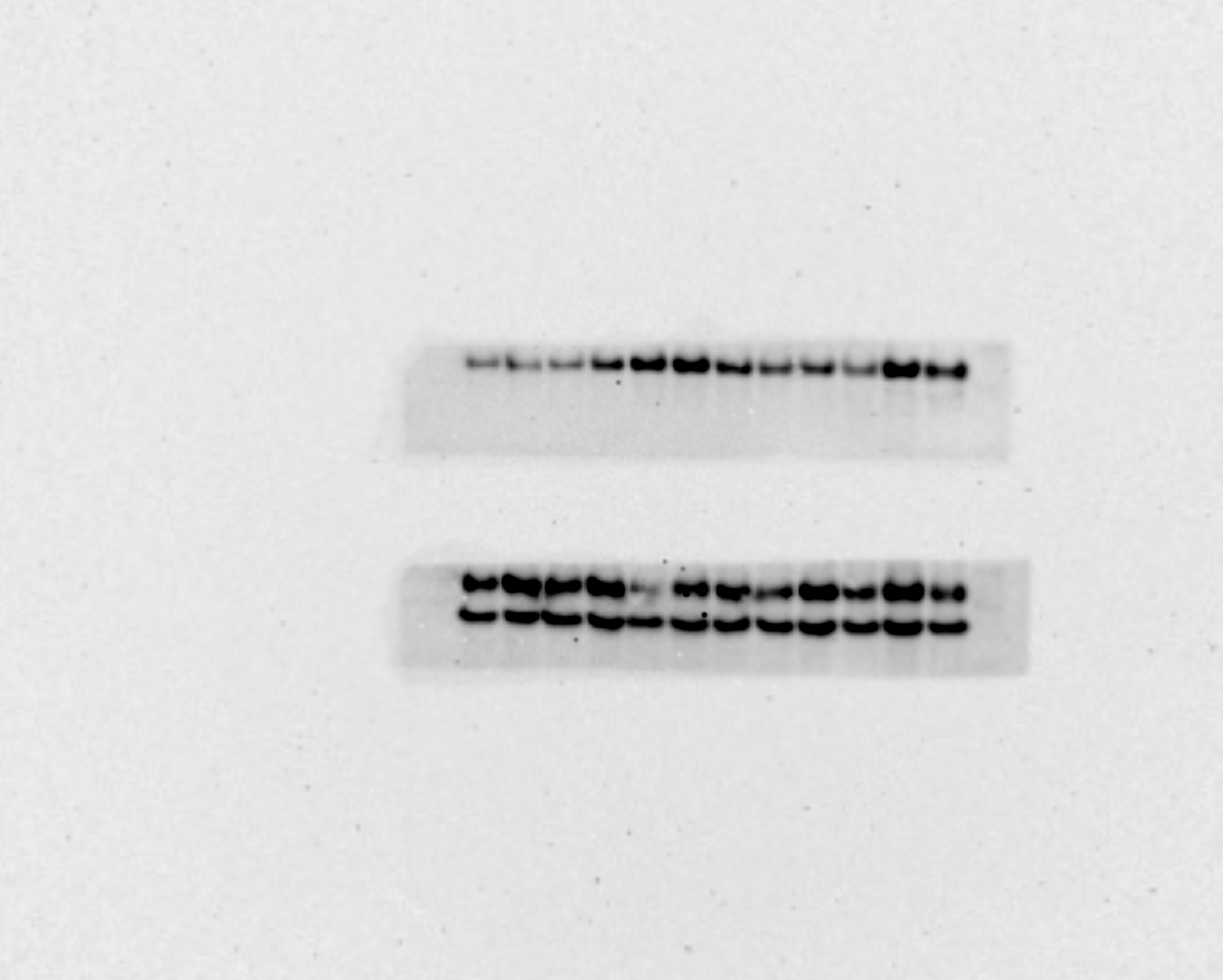

Supplement: Figure 5—source data 2. [file elife-89509-fig5-data2.zip › Figure 5-Source data 1/Figure 5-Source data 1 for Figure 5J-Unedited Blots-pp65+p65 (ST+TPEN).tif]

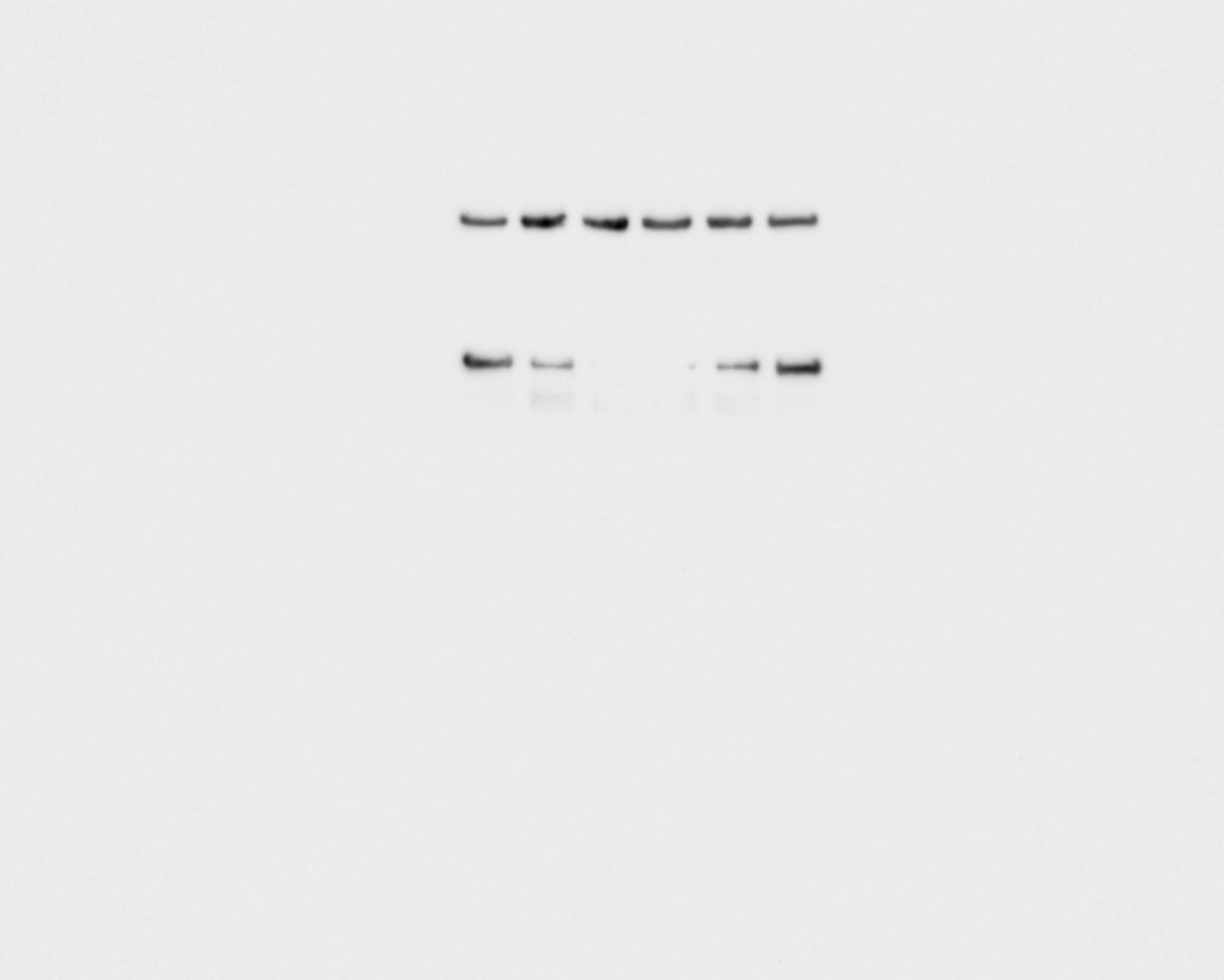

Supplement: Figure 5—source data 2. [file elife-89509-fig5-data2.zip › Figure 5-Source data 1/Figure 5-Source data 1 for Figure 5H-Unedited Blots-p65 (ST).tif]

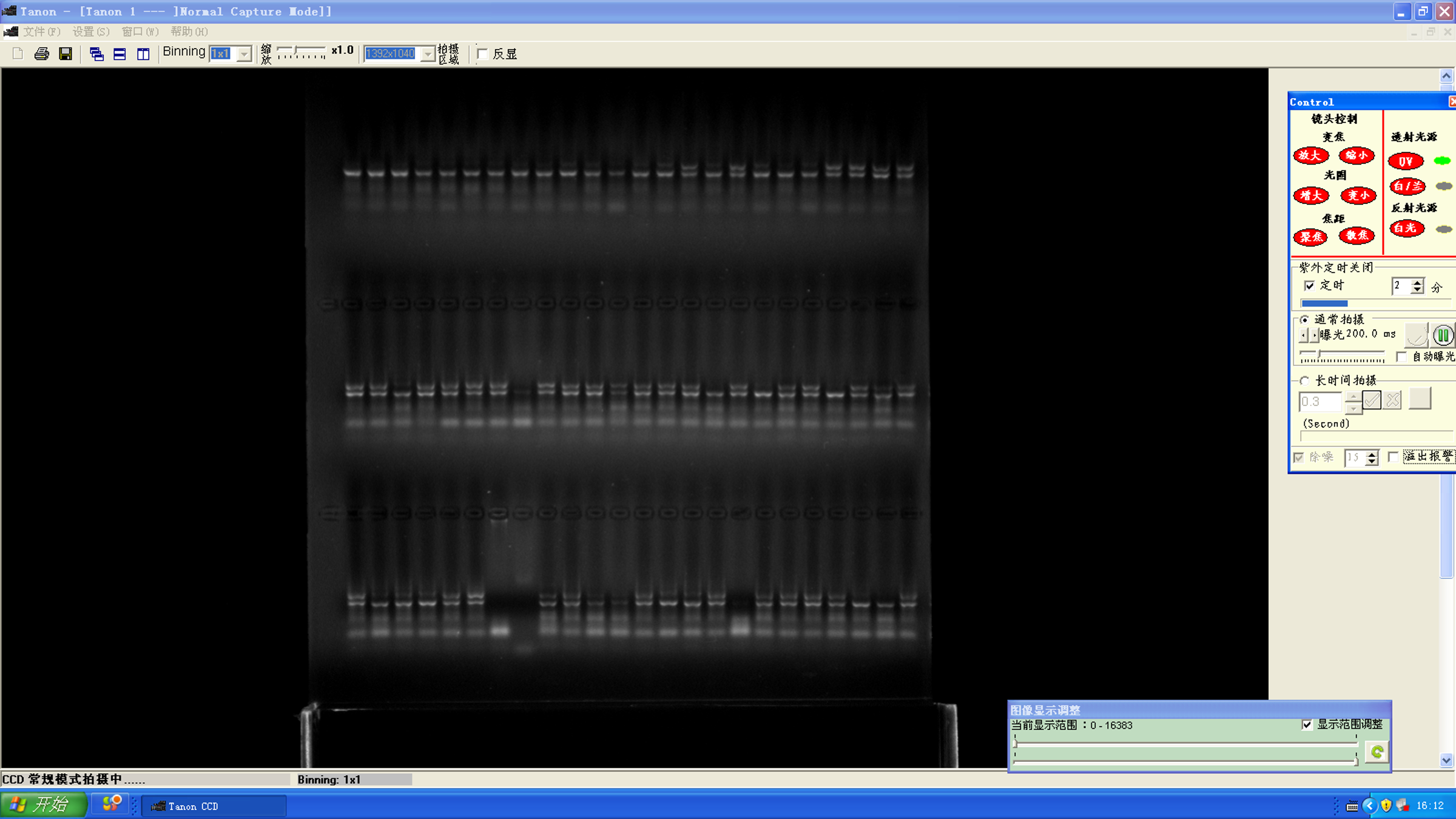

Supplement: Figure 8—source data 1. [file elife-89509-fig8-data1.zip › Figure 8-Source data 1/Figure supplement 1-Source data 1-Unedited gel-PCR Lyz2 Cre.png]

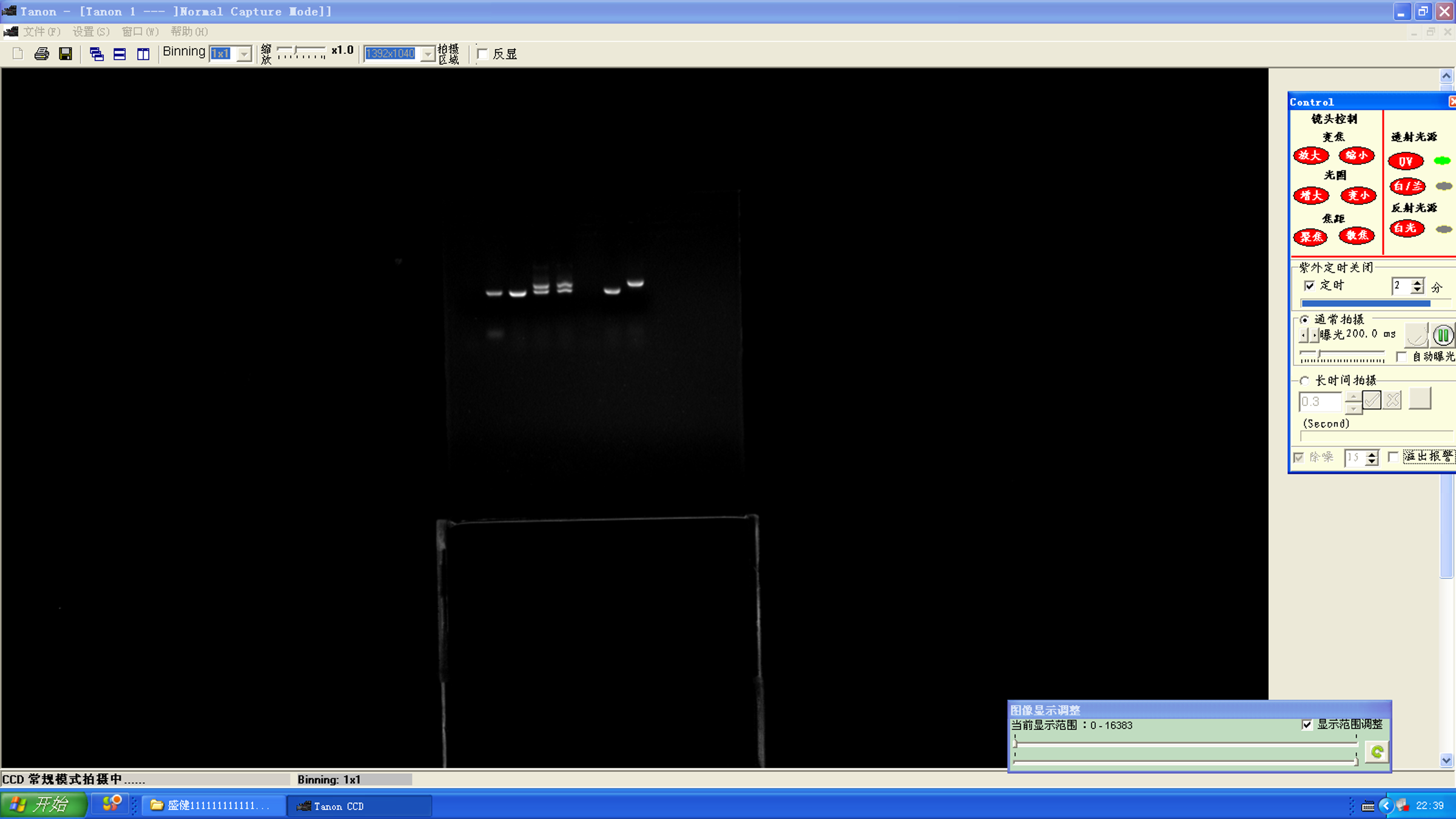

Supplement: Figure 8—source data 1. [file elife-89509-fig8-data1.zip › Figure 8-Source data 1/Figure supplement 1-Source data 1-Unedited gel-PCR genotype Slc30a1 flox.png]

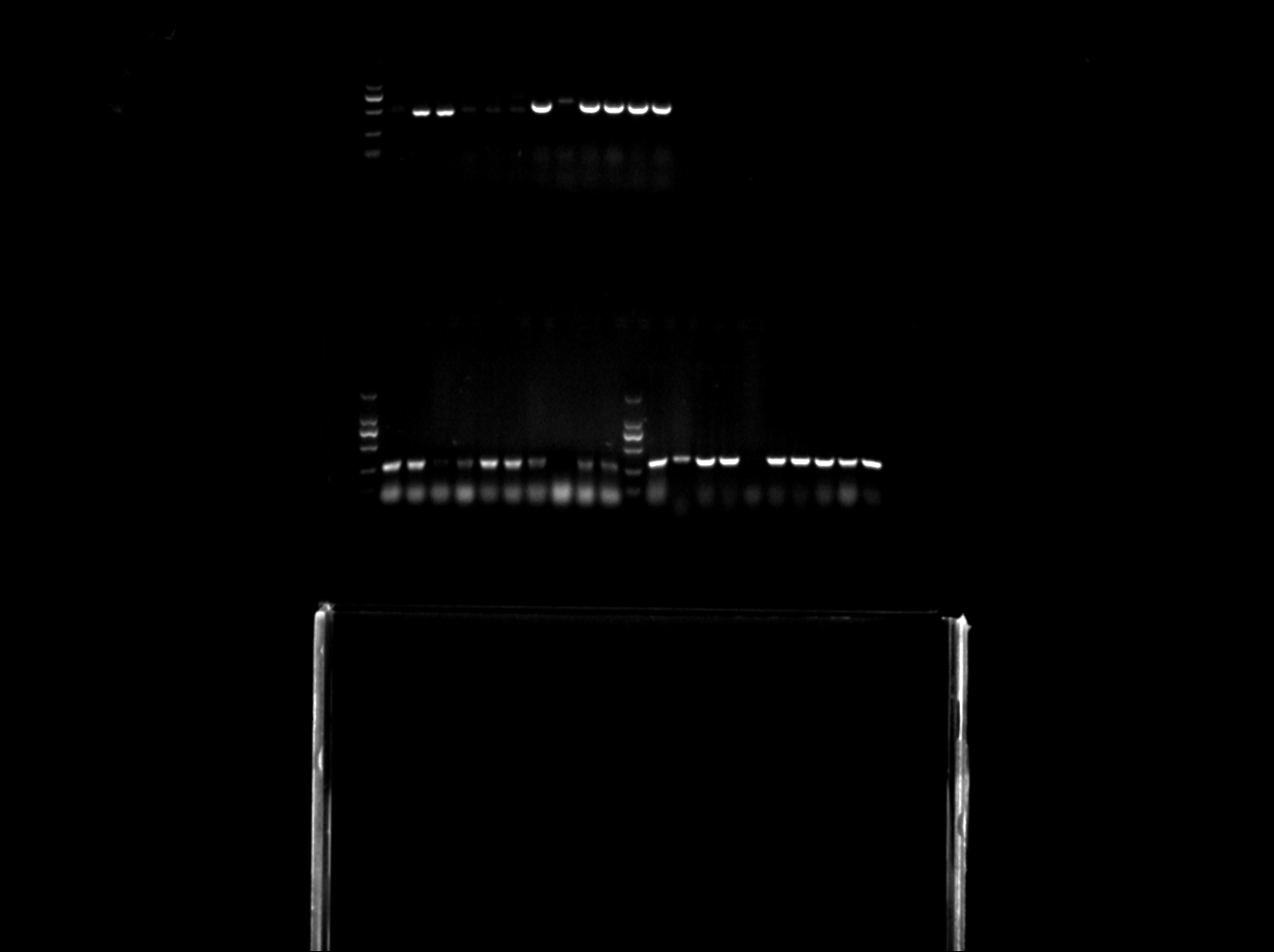

Supplement: Figure 8—source data 1. [file elife-89509-fig8-data1.zip › Figure 8-Source data 1/Figure supplement 1-Source data 1-Unedited gel-PCR Slc30a1-Flag-EGFP.png]
