# Supplementary material for: The zinc transporter Slc30a1 (ZnT1) in macrophages plays a protective role against attenuated Salmonella
Source: eLife. 2024 Oct 30;13:e89509. doi: 10.7554/eLife.89509 (PMC11524588; doi:10.7554/eLife.89509)
Supplement: Figure 5—source data 1. [file elife-89509-fig5-data1.zip › Figure 5-Figure supplement 1-Source data 2/Figure 5-figure supplement 1-Source data 2_Raw images of western blot analysis for iNOS and related-cellular signaling proteins.pdf]

Figure 5–Figure supplement 1–Source data 1
Raw images of western blot analysis for iNOS and related-cellular signaling proteins.

Full unedited blots for Figure supplement 6C (iNOS expression in BMDM treated with HK-ST)

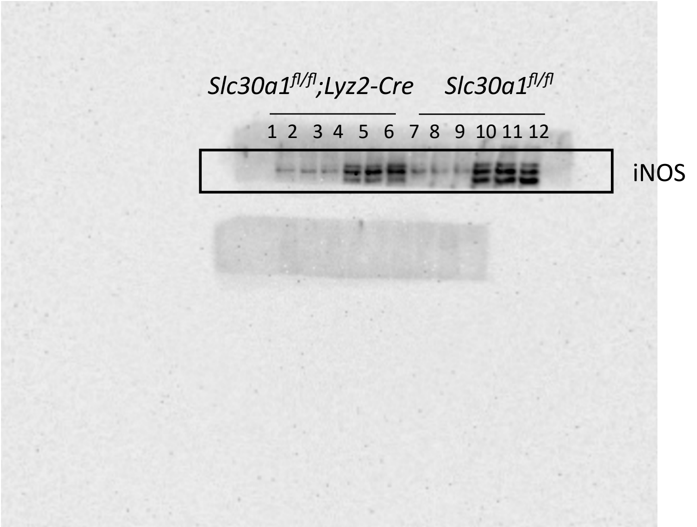

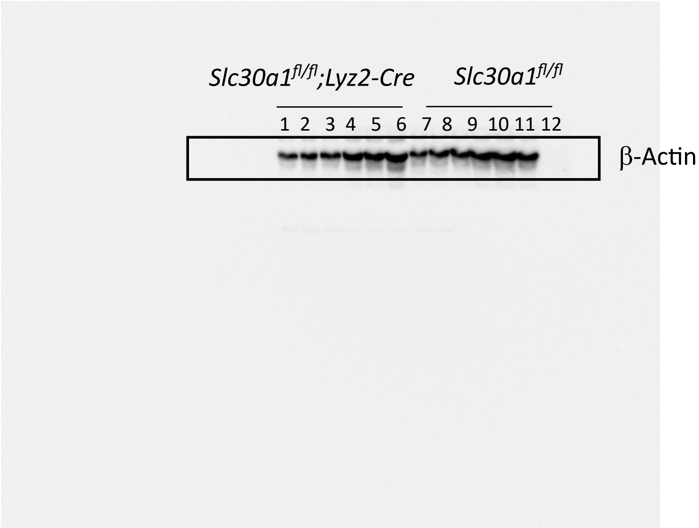

Lane 1, 2, 3: Untreated
Lane 4, 5, 6: HK-ST
| *Slc30a1*$^{fl/fl}$*;Lyz2-Cre*

Lane 7, 8, 9: Untreated
Lane 10, 11, 12: HK-ST
| *Slc30a1*$^{fl/fl}$

Full unedited blots for Figure supplement 6D (iNOS expression in BMDM treated with LPS)

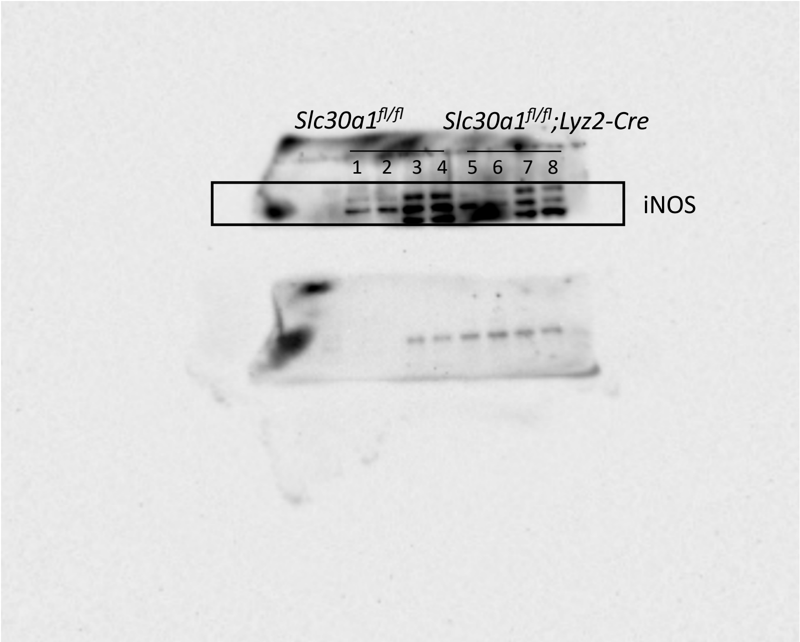

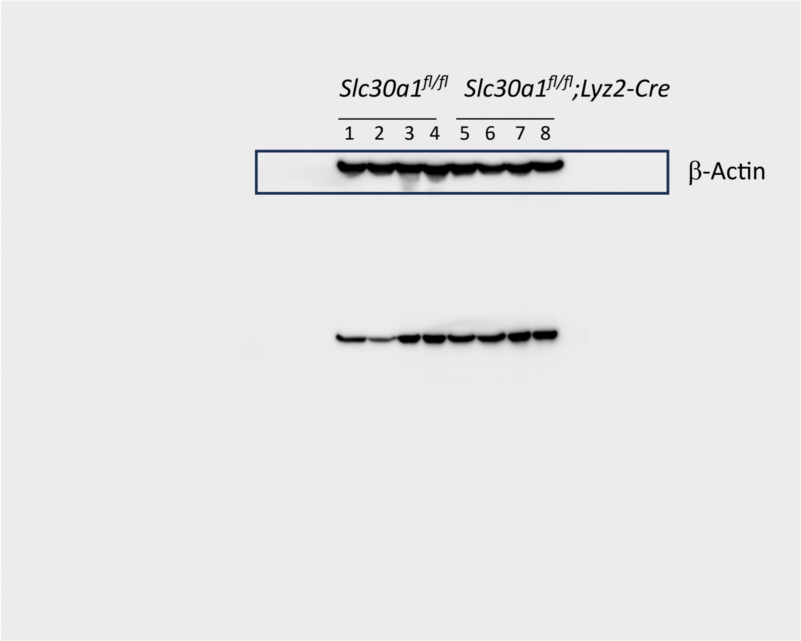

Lane 1, 2: Untreated
Lane 3, 4: LPS

Slc30a1$^{fl/fl}$

Lane 5, 6: Untreated
Lane 7, 8: LPS

Slc30a1$^{fl/fl}$*;Lyz2-Cre*

Full unedited blots for Figure supplement 6F (cellular signaling proteins regulated *Nos2* expression in BMDM treated with LPS)

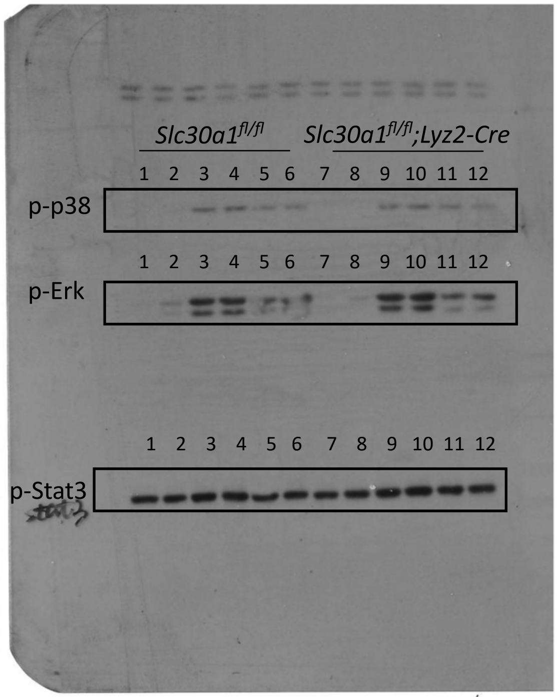

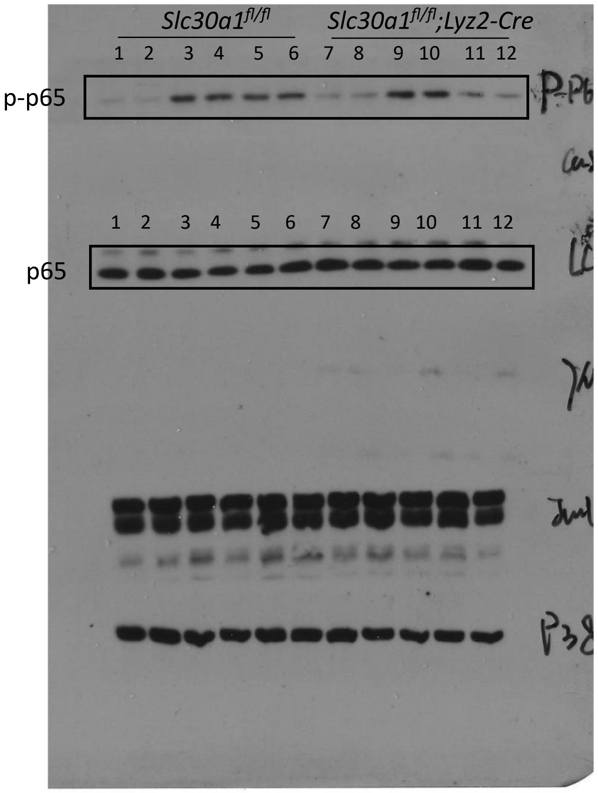

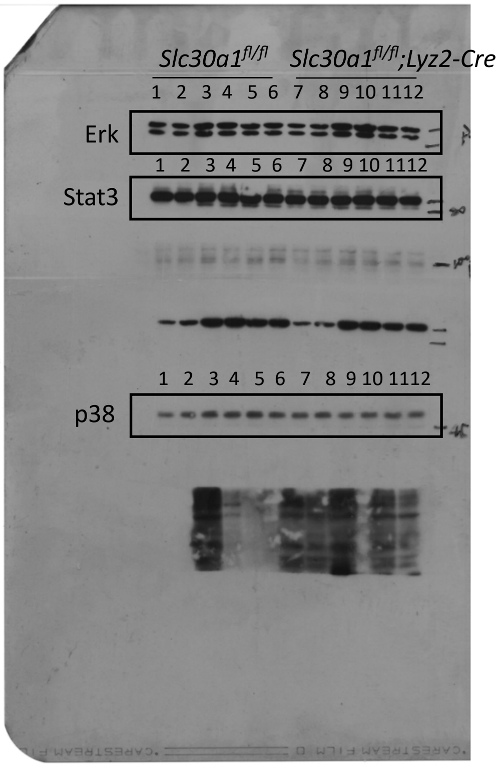

Lane 1, 2: Untreated		*Slc30a1*$^{fl/fl}$
Lane 3, 4: LPS (30 min)		
Lane 5, 6: LPS (60 min)		
Lane 7, 8: Untreated		*Slc30a1*$^{fl/fl}$*;Lyz2-Cre*
Lane 9, 10: LPS (30 min)		
Lane 11, 12: LPS (60 min)		
