# Supplementary material for: The zinc transporter Slc30a1 (ZnT1) in macrophages plays a protective role against attenuated Salmonella
Source: eLife. 2024 Oct 30;13:e89509. doi: 10.7554/eLife.89509 (PMC11524588; doi:10.7554/eLife.89509)
Supplement: Figure 5—source data 2. [file elife-89509-fig5-data2.zip › Figure 5-Source data 1/Figure 5-Source data 1_Raw images of western blot analysis for iNOS and p65 expression.pdf]

Figure 5–Source data 1
Raw images of western blot analysis for iNOS and p65 expression.

Full unedited blots for Figure 5F (iNOS expression in BMDM infected with *Salmonella*)

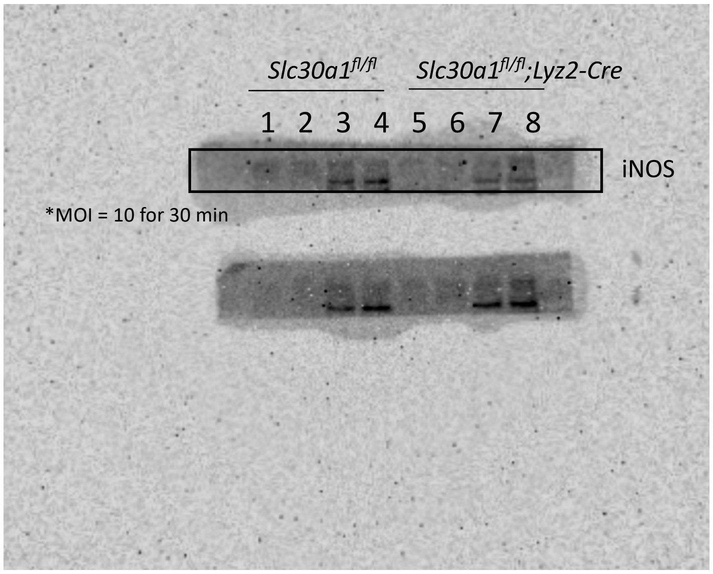

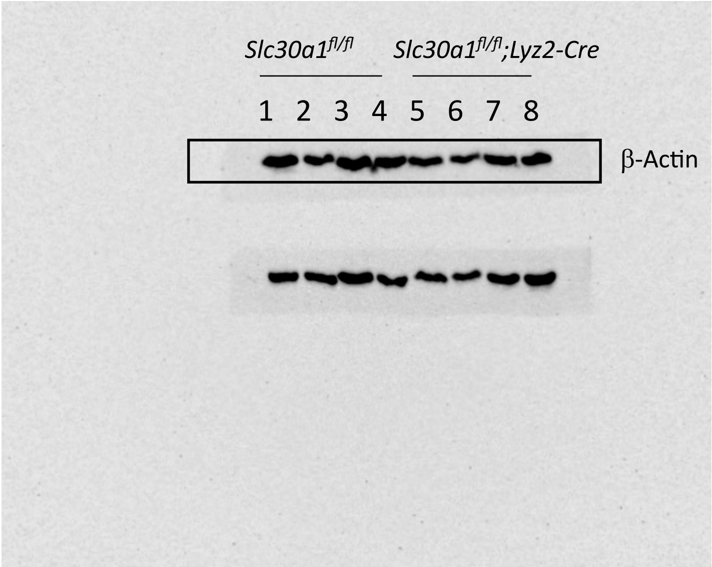

Lane 1, 2: Untreated
Lane 3, 4: *Salmonella*
Slc30a1$^{fl/fl}$

Lane 5, 6: Untreated
Lane 7, 8: *Salmonella*
Slc30a1$^{fl/fl}$*;Lyz2-Cre*

Full unedited blots for Figure 5H (p65 expression in BMDM infected with *Salmonella*)

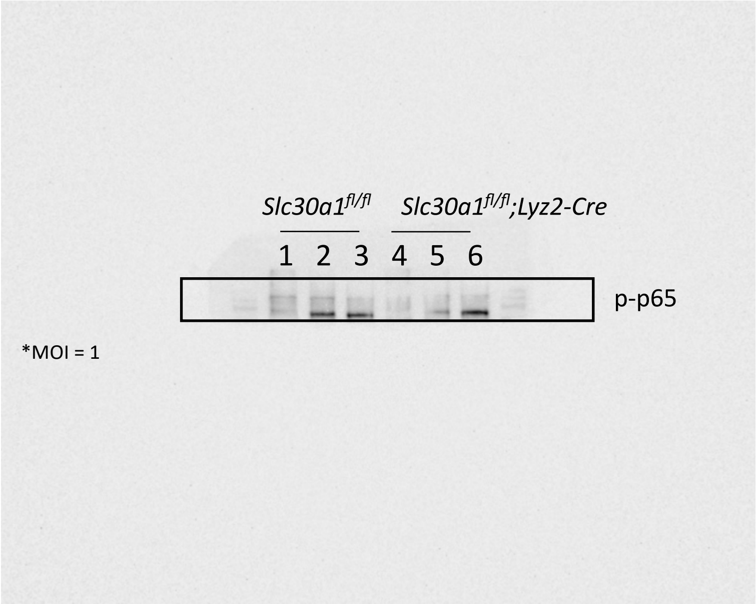

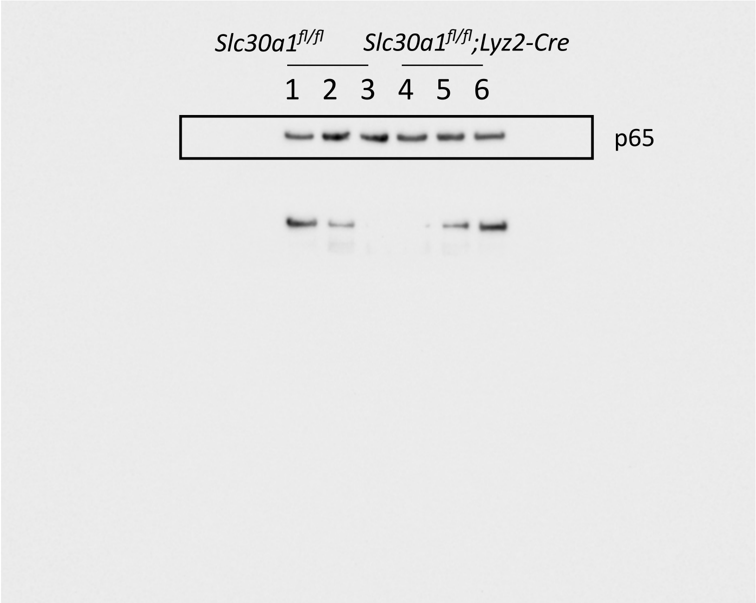

Lane 1: Uninfected
Lane 2: *Salmonella* (1 h)
Lane 3: *Salmonella* (2 h)

Slc30a1$^{fl/fl}$

Lane 4: Uninfected
Lane 5: *Salmonella* (1 h)
Lane 6: *Salmonella* (2 h)

Slc30a1$^{fl/fl}$*;Lyz2-Cre*

Full unedited blots for Figure 5J (p65 expression in BMDM treated with *Salmonella* plus TPEN)

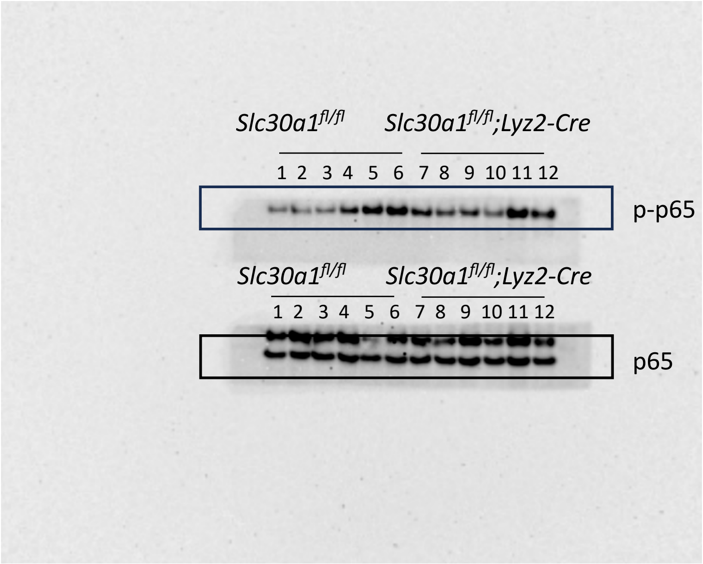

Lane 1, 2: Uninfected<br>Lane 3, 4: TPEN<br>Lane 5, 6: TPEN + *Salmonella*	*Slc30a1$^{fl/fl}$*
Lane 7, 8: Uninfected<br>Lane 9, 10: TPEN<br>Lane 11, 12: TPEN + *Salmonella*	*Slc30a1$^{fl/fl}$;Lyz2-Cre*
