# Supplementary material for: The zinc transporter Slc30a1 (ZnT1) in macrophages plays a protective role against attenuated Salmonella
Source: eLife. 2024 Oct 30;13:e89509. doi: 10.7554/eLife.89509 (PMC11524588; doi:10.7554/eLife.89509)
Supplement: Figure 8—source data 1. [file elife-89509-fig8-data1.zip › Figure 8-Source data 1/Figure 8-Source data 1.pdf]

Figure 8–Source data 1. Raw images of PCR genotype analysis

Full unedited gel for figure supplement 9B (*Slc30a1-flag-EGFP*)

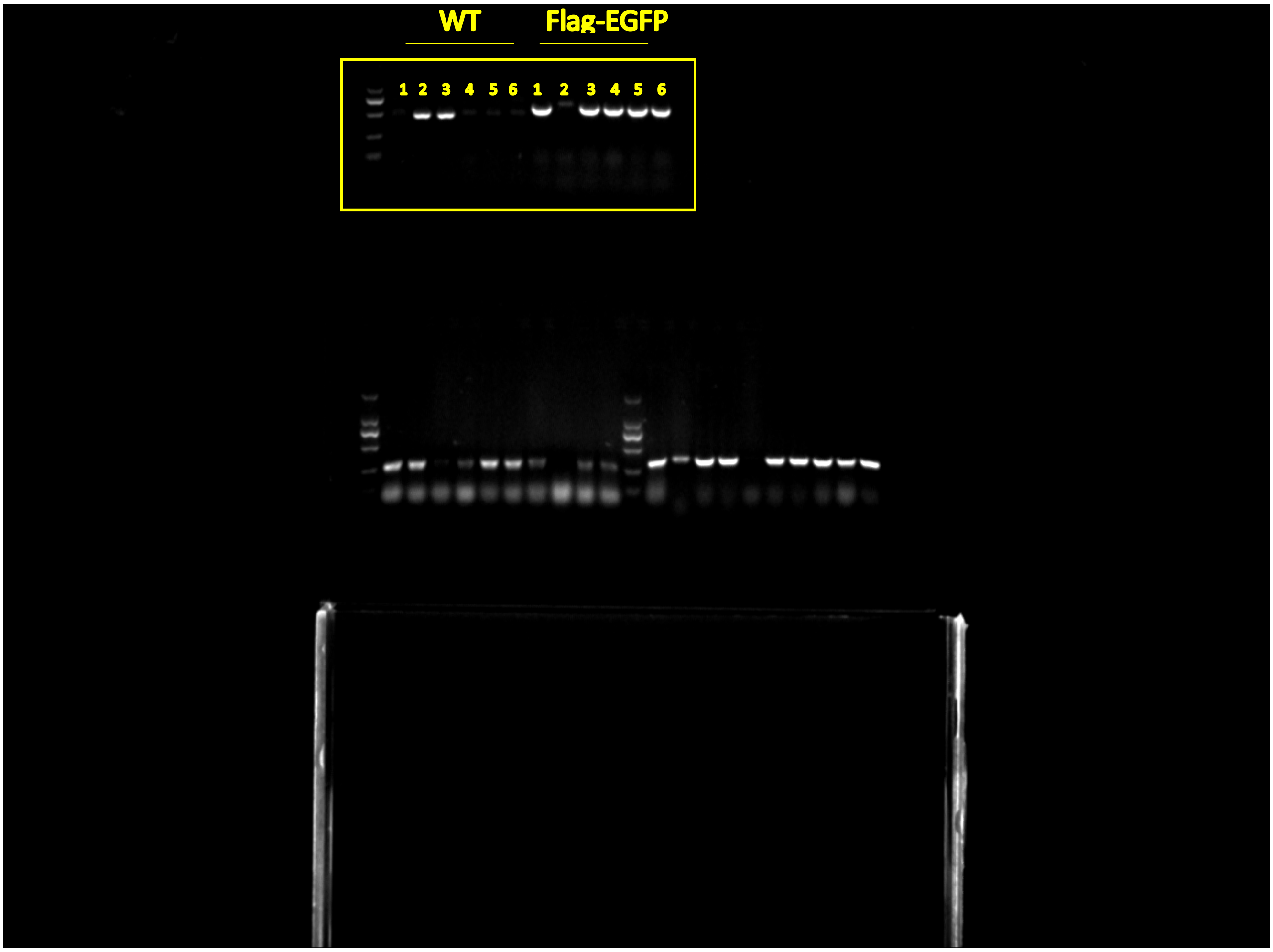

WT Lane 1: Mutant
Lane 2: Wild type
Lane 3: Wild type
Lane 4: Mutant
Lane 5: Mutant

Flag-EGFP Lane 1: Mutant
Lane 2: Wile type
Lane 3: Mutant
Lane 4: Mutant
Lane 5: Mutant
Lane 6: Mutant

Input: Lane 1, 4, 5, 6 – $Slc30a1^{flag\text{-}EGFP/flag\text{-}EGFP}$, Lane 3 – $Slc30a1^{flag\text{-}EGFP/+}$, Lane 2 – Wild type

Full unedited gel for figure supplement 9C (*Slc30a1-flox*)

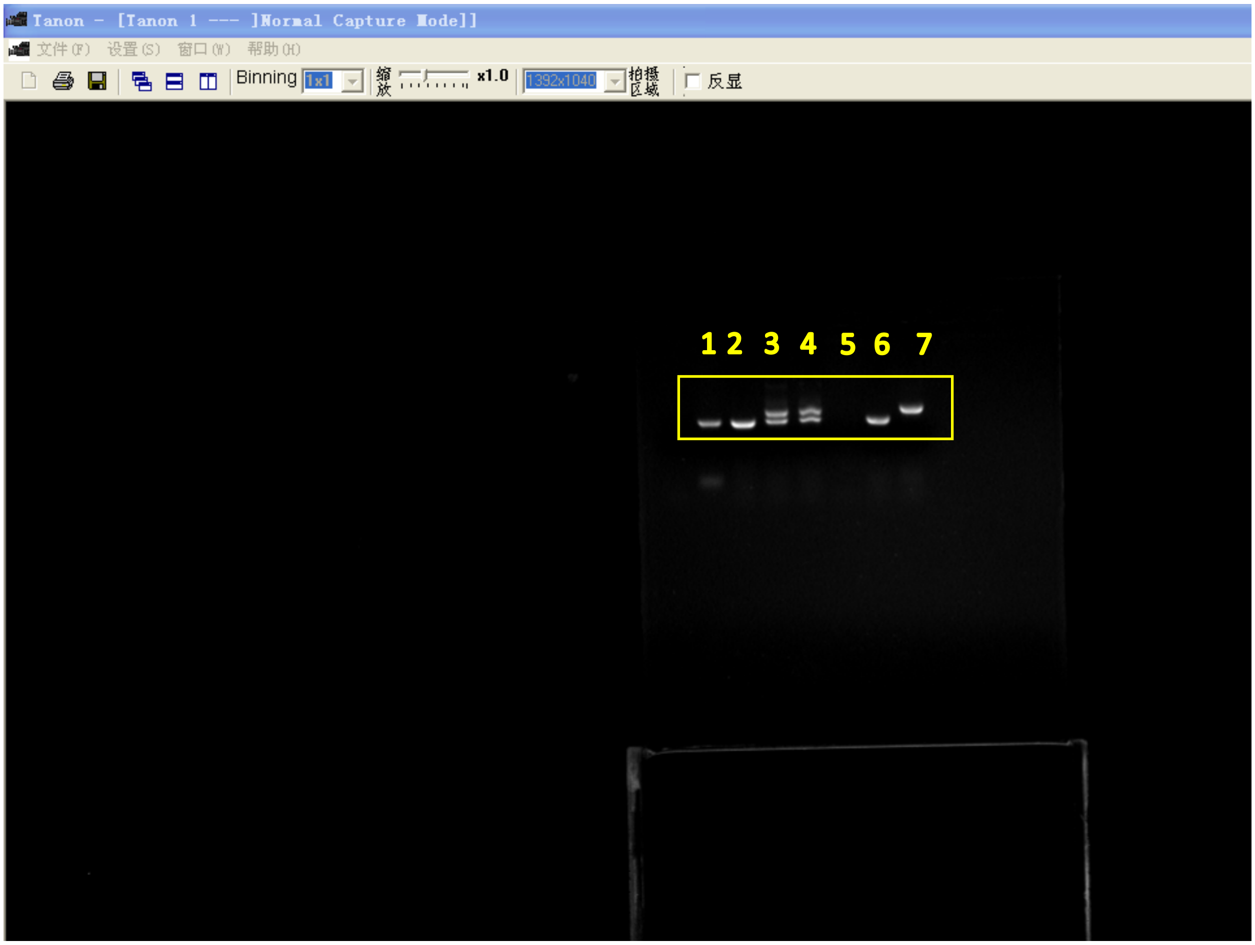

Lane 1: Wild type
Lane 2: Wild type
Lane 3: *Slc30a1*$^{fl/+}$
Lane 4: *Slc30a1*$^{fl/+}$
Lane 5: Blank
Lane 6: Wild type
Lane 7: *Slc30a1*$^{fl/fl}$

Full unedited gel for figure supplement 9C (*Lyz2* Cre)

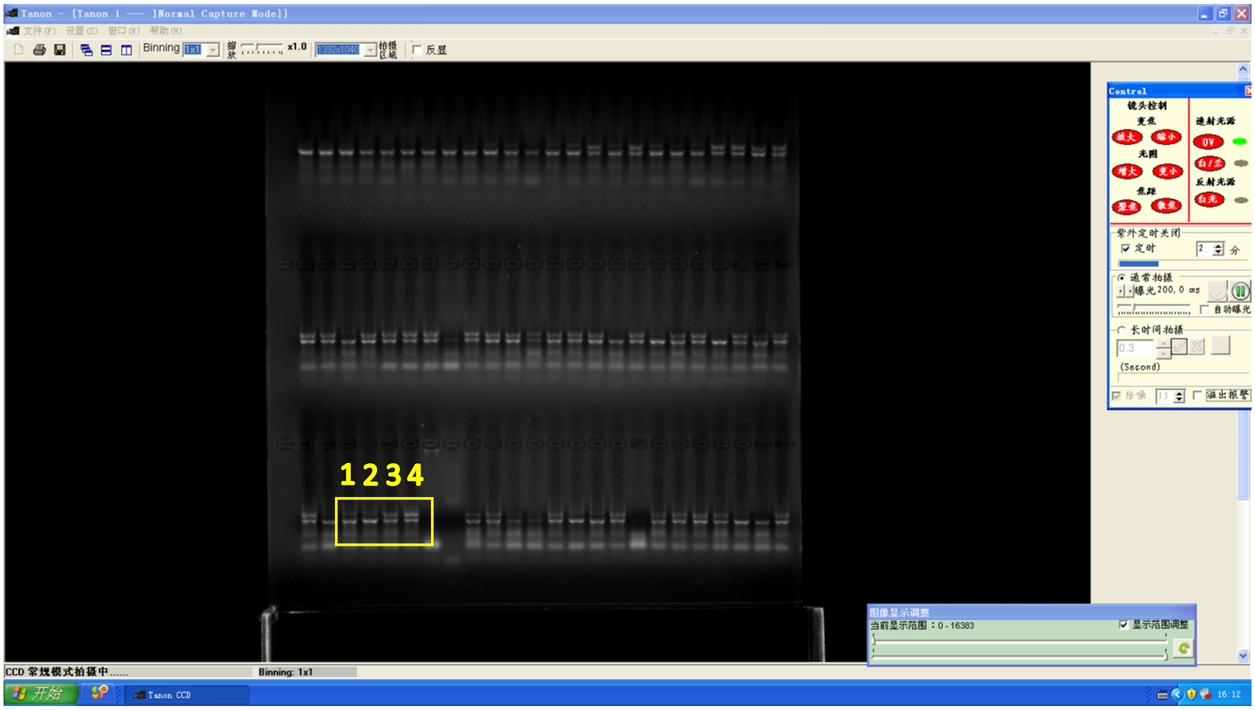

Lane 1: Wild type

Lane 2: Wild type

Lane 3: *Lyz2* Cre$^{+/-}$

Lane 4: *Lyz2* Cre$^{+/-}$
