# Supplementary material for: The zinc transporter Slc30a1 (ZnT1) in macrophages plays a protective role against attenuated Salmonella
Source: eLife. 2024 Oct 30;13:e89509. doi: 10.7554/eLife.89509 (PMC11524588; doi:10.7554/eLife.89509)
Supplement: Supplementary file 1. [file elife-89509-supp1.docx]

**Supplementary file 1.** Top 50 enriched GO terms for the DEGs in C57BL/6 BMDMs infected with *Salmonella* versus uninfected cells.

|  | GO ID | Term | Function | *P*-value |
| --- | --- | --- | --- | --- |
| 1 | GO:0005515 | Protein binding | Molecular function | 2.31E-14 |
| 2 | GO:0003676 | Nucleic acid binding | Molecular function | 1.65E-11 |
| 3 | GO:0046872 | Metal ion binding | Molecular function | 2.02E-11 |
| 4 | GO:0000122 | Negative regulation of transcription by RNA polymerase II | Biological process | 1.77E-08 |
| 5 | GO:0006355 | Regulation of transcription, DNA-templated | Biological process | 2.08E-08 |
| 6 | GO:0003700 | DNA-binding transcription factor activity | Molecular function | 4.02E-08 |
| 7 | GO:0005737 | Cytoplasm | Cellular component | 5.18E-08 |
| 8 | GO:0045944 | Positive regulation of transcription by RNA polymerase II | Biological process | 7.75E-08 |
| 9 | GO:0010575 | Positive regulation of vascular endothelial growth factor production | Biological process | 1.12E-07 |
| 10 | GO:0005829 | Cytosol | Cellular component | 1.36E-07 |
| 11 | GO:0045893 | Positive regulation of transcription, DNA-templated | Biological process | 6.28E-07 |
| 12 | GO:0005654 | Nucleoplasm | Cellular component | 7.75E-07 |
| 13 | GO:0005634 | Nucleus | Cellular component | 8.66E-07 |
| 14 | GO:0032755 | Positive regulation of interleukin-6 production | Biological process | 4.31E-06 |
| 15 | GO:0000978 | RNA polymerase II proximal promoter sequence-specific DNA binding | Molecular function | 5.41E-06 |
| 16 | GO:0003677 | DNA binding | Molecular function | 1.27E-05 |
| 17 | GO:0043154 | Negative regulation of cysteine-type endopeptidase activity involved in apoptotic process | Biological process | 1.27E-05 |
| 18 | GO:0007179 | Transforming growth factor beta receptor signaling pathway | Biological process | 1.45E-05 |
| 19 | GO:0006954 | **Inflammatory response** | Biological process | 1.73E-05 |
| 20 | GO:0032496 | **Response to lipopolysaccharide** | Biological process | 1.89E-05 |
| 21 | GO:0032757 | Positive regulation of interleukin-8 production | Biological process | 1.97E-05 |
| 22 | GO:0071230 | Cellular response to amino acid stimulus | Biological process | 2.06E-05 |
| 23 | GO:0003713 | Transcription coactivator activity | Molecular function | 2.17E-05 |
| 24 | GO:0002196 | Ser-tRNA(Ala) hydrolase activity | Molecular function | 3.97E-05 |
| 25 | GO:0043507 | Positive regulation of JUN kinase activity | Biological process | 4.30E-05 |
| 26 | GO:0008285 | Negative regulation of cell population proliferation | Biological process | 4.94E-05 |
| 27 | GO:0005623 | Cell | Cellular component | 5.17E-05 |
| 28 | GO:0043392 | Negative regulation of DNA binding | Biological process | 5.41E-05 |
| 29 | GO:0016477 | Cell migration | Biological process | 6.52E-05 |
| 30 | GO:0002237 | **Response to molecule of bacterial origin** | Biological process | 7.11E-05 |
| 31 | GO:0001568 | Blood vessel development | Biological process | 9.90E-05 |
| 32 | GO:0005085 | Guanyl-nucleotide exchange factor activity | Molecular function | 1.34E-04 |
| 33 | GO:0043433 | Negative regulation of DNA-binding transcription factor activity | Biological process | 1.43E-04 |
| 34 | GO:0032725 | Positive regulation of granulocyte macrophage colony-stimulating factor production | Biological process | 1.48E-04 |
| 35 | GO:0006419 | Alanyl-tRNA aminoacylation | Biological process | 1.55E-04 |
| 36 | GO:0004813 | Alanine-tRNA ligase activity | Molecular function | 1.55E-04 |
| 37 | GO:0010906 | Regulation of glucose metabolic process | Biological process | 1.58E-04 |
| 38 | GO:0045892 | Negative regulation of transcription, DNA-templated | Biological process | 1.62E-04 |
| 39 | GO:0045785 | Positive regulation of cell adhesion | Biological process | 1.65E-04 |
| 40 | GO:0007264 | Small GTPase mediated signal transduction | Biological process | 1.71E-04 |
| 41 | GO:0032088 | Negative regulation of NF-kappaB transcription factor activity | Biological process | 1.81E-04 |
| 42 | GO:0071222 | **Cellular response to lipopolysaccharide** | Biological process | 1.84E-04 |
| 43 | GO:0043122 | Regulation of I-kappaB kinase/NF-kappaB signaling | Biological process | 2.00E-04 |
| 44 | GO:0032729 | Positive regulation of interferon-gamma production | Biological process | 2.01E-04 |
| 45 | GO:1904262 | Negative regulation of TORC1 signaling | Biological process | 2.02E-04 |
| 46 | GO:0032869 | Cellular response to insulin stimulus | Biological process | 2.26E-04 |
| 47 | GO:0043177 | Organic acid binding | Molecular function | 2.41E-04 |
| 48 | GO:1904996 | Positive regulation of leukocyte adhesion to vascular endothelial cell | Biological process | 2.41E-04 |
| 49 | GO:0090023 | Positive regulation of neutrophil chemotaxis | Biological process | 2.50E-04 |
| 50 | GO:0035556 | Intracellular signal transduction | Biological process | 2.68E-04 |
