# Supplementary material for: The zinc transporter Slc30a1 (ZnT1) in macrophages plays a protective role against attenuated Salmonella
Source: eLife. 2024 Oct 30;13:e89509. doi: 10.7554/eLife.89509 (PMC11524588; doi:10.7554/eLife.89509)
Supplement: Supplementary file 2. [file elife-89509-supp2.docx]

**Supplementary file 2.** KEGG enrichment pathways of the DEGs in C57BL/6 BMDMs infected with *Salmonella* versus uninfected cells.

|  | Pathway ID | Pathway name | *P*-value | Rich factor | Class |
| --- | --- | --- | --- | --- | --- |
| 1 | ko04668 | TNF signaling pathway | 1.27E-06 | 0.17 | Environmental Information Processing |
| 2 | ko05152 | Tuberculosis | 5.60E-05 | 0.12 | Human Diseases |
| 3 | ko05168 | Herpes simplex virus 1 infection | 1.79E-04 | 0.09 | Human Diseases |
| 4 | ko04625 | C-type lectin receptor signaling pathway | 1.93E-04 | 0.13 | Organismal Systems |
| 5 | ko05144 | Malaria | 2.16E-04 | 0.18 | Human Diseases |
| 6 | ko04657 | IL-17 signaling pathway | 2.26E-04 | 0.14 | Organismal Systems |
| 7 | ko05143 | African trypanosomiasis | 4.66E-04 | 0.21 | Human Diseases |
| 8 | ko05202 | Transcriptional misregulation in cancer | 5.63E-04 | 0.10 | Human Diseases |
| 9 | ko04620 | Toll-like receptor signaling pathway | 9.64E-04 | 0.13 | Organismal Systems |
| 10 | ko05161 | Hepatitis B | 1.29E-03 | 0.10 | Human Diseases |
| 11 | ko04064 | NF-kappa B signaling pathway | 1.41E-03 | 0.12 | Environmental Information Processing |
| 12 | ko05321 | Inflammatory bowel disease (IBD) | 2.06E-03 | 0.15 | Human Diseases |
| 13 | ko04933 | AGE-RAGE signaling pathway in diabetic complications | 2.81E-03 | 0.11 | Human Diseases |
| 14 | ko05146 | Amoebiasis | 3.05E-03 | 0.11 | Human Diseases |
| 15 | ko05162 | Measles | 3.17E-03 | 0.10 | Human Diseases |
| 16 | ko04660 | T cell receptor signaling pathway | 3.56E-03 | 0.11 | Organismal Systems |
| 17 | ko04218 | Cellular senescence | 3.60E-03 | 0.09 | Cellular Processes |
| 18 | ko04150 | mTOR signaling pathway | 4.25E-03 | 0.09 | Environmental Information Processing |
| 19 | ko04380 | Osteoclast differentiation | 4.81E-03 | 0.10 | Organismal Systems |
| 20 | ko04024 | cAMP signaling pathway | 5.90E-03 | 0.08 | Environmental Information Processing |
| 21 | ko05142 | Chagas disease (American trypanosomiasis) | 7.33E-03 | 0.10 | Human Diseases |
| 22 | ko04060 | Cytokine-cytokine receptor interaction | 7.47E-03 | 0.08 | Environmental Information Processing |
| 23 | ko04015 | Rap1 signaling pathway | 8.62E-03 | 0.08 | Environmental Information Processing |
| 24 | ko04550 | Signaling pathways regulating pluripotency of stem cells | 9.57E-03 | 0.09 | Cellular Processes |
| 25 | ko05222 | Small cell lung cancer | 1.12E-02 | 0.10 | Human Diseases |
| 26 | ko04350 | TGF-beta signaling pathway | 1.12E-02 | 0.10 | Environmental Information Processing |
| 27 | ko04611 | Platelet activation | 1.21E-02 | 0.09 | Organismal Systems |
| 28 | ko05332 | Graft-versus-host disease | 1.25E-02 | 0.13 | Human Diseases |
| 29 | ko04930 | Type II diabetes mellitus | 1.39E-02 | 0.13 | Human Diseases |
| 30 | ko05418 | Fluid shear stress and atherosclerosis | 1.41E-02 | 0.09 | Human Diseases |
| 31 | ko05140 | Leishmaniasis | 1.68E-02 | 0.11 | Human Diseases |
| 32 | ko04210 | Apoptosis | 1.70E-02 | 0.09 | Cellular Processes |
| 33 | ko05166 | Human T-cell leukemia virus 1 infection | 1.81E-02 | 0.08 | Human Diseases |
| 34 | ko05323 | Rheumatoid arthritis | 2.11E-02 | 0.10 | Human Diseases |
| 35 | ko05134 | Legionellosis | 2.21E-02 | 0.12 | Human Diseases |
| 36 | ko05167 | Kaposi sarcoma-associated herpesvirus infection | 2.26E-02 | 0.08 | Human Diseases |
| 37 | ko00970 | Aminoacyl-tRNA biosynthesis | 2.36E-02 | 0.13 | Genetic Information Processing |
| 38 | ko04659 | Th17 cell differentiation | 2.44E-02 | 0.09 | Organismal Systems |
| 39 | ko05163 | Human cytomegalovirus infection | 2.47E-02 | 0.07 | Human Diseases |
| 40 | ko04010 | MAPK signaling pathway | 2.54E-02 | 0.07 | Environmental Information Processing |
| 41 | ko04932 | Non-alcoholic fatty liver disease (NAFLD) | 2.69E-02 | 0.08 | Human Diseases |
| 42 | ko04914 | Progesterone-mediated oocyte maturation | 2.74E-02 | 0.09 | Organismal Systems |
| 43 | ko05133 | Pertussis | 2.84E-02 | 0.10 | Human Diseases |
| 44 | ko04211 | Longevity regulating pathway | 2.91E-02 | 0.09 | Organismal Systems |
| 45 | ko04621 | NOD-like receptor signaling pathway | 2.98E-02 | 0.08 | Organismal Systems |
| 46 | ko04370 | VEGF signaling pathway | 3.06E-02 | 0.11 | Environmental Information Processing |
| 47 | ko04931 | Insulin resistance | 3.22E-02 | 0.09 | Human Diseases |
| 48 | ko05132 | *Salmonella* infection | 3.25E-02 | 0.10 | Human Diseases |
| 49 | ko04926 | Relaxin signaling pathway | 3.27E-02 | 0.08 | Organismal Systems |
| 50 | ko01523 | Antifolate resistance | 3.29E-02 | 0.14 | Human Diseases |
| 51 | ko05205 | Proteoglycans in cancer | 3.63E-02 | 0.07 | Human Diseases |
| 52 | ko04978 | Mineral absorption | 3.76E-02 | 0.11 | Organismal Systems |
| 53 | ko04640 | Hematopoietic cell lineage | 3.90E-02 | 0.09 | Organismal Systems |
| 54 | ko04630 | Jak-STAT signaling pathway | 4.42E-02 | 0.08 | Environmental Information Processing |
