# Supplementary material for: The zinc transporter Slc30a1 (ZnT1) in macrophages plays a protective role against attenuated Salmonella
Source: eLife. 2024 Oct 30;13:e89509. doi: 10.7554/eLife.89509 (PMC11524588; doi:10.7554/eLife.89509)
Supplement: Supplementary file 3. [file elife-89509-supp3.docx]

**Supplementary file 3.** Summary of blood test results for uninfected and *Salmonella*-infected *Slc30a1^fl/fl^* and *Slc30a1^fl/fl^;Lyz2-Cre* mice.

| Measurement | *Slc30a1^fl/fl^*  Uninfected (*n* = 5) | *Slc30a1^fl/fl^;Lyz2-Cre*  Uninfected (*n* = 6) | *Slc30a1^fl/fl^*  24 hpi (*n* = 5) | *Slc30a1^fl/fl^;Lyz2-Cre*  24 hpi (*n* = 4) |
| --- | --- | --- | --- | --- |
| WBC (×10^9^/L) | 2.29±0.57 | 1.89±0.33 | 2.46±1.01 | 1.38±0.53 |
| RBC (×10^12^/L) | 9.02±0.29 | 8.85±0.57 | 9.07±0.63 | 8.72±0.36 |
| Hb (g/dL) | 13.46±0.34 | 13.37±0.70 | 12.82±1.10 | 12.53±0.44 |
| Hct (%) | 43.56±1.45 | 43.42±2.19 | 40.98±2.99 | 40.68±1.19 |
| MCV (fL) | 48.26±0.84 | 49.08±1.15 | 45.22±1.96 | 46.63±0.59 |
| MCH (pg) | 14.94±0.25 | 15.12±0.22 | 14.14±0.52 | 14.35±0.06 |
| MCHC (g/L) | 30.90±0.41 | 30.80±0.52 | 31.26±0.67 | 30.80±0.22 |
| PLT (×10^9^/L) | 1301 ±111 | 1086±210 | 717±99 | 788±148 |
| LYM (×10^9^/L) | 1.61±0.42 | 1.44±0.09 | 1.45±0.40 | 0.77±0.25* |
| NEUT (×10^9^/L) | 0.47±0.15 | 0.22±0.07** | 0.59±0.42 | 0.37±0.19 |
| MONO (×10^9^/L) | 0.16±0.05 | 0.10±0.03* | 0.51±0.19 | 0.20±0.07* |
| EO (×10^9^/L) | 0.04±0.01 | 0.02±0.02 | 0.04±0.03 | 0.02±0.01 |
| BASO (×10^9^/L) | 0.00±0.01 | 0.00±0.00 | 0.05±0.11 | 0.02±0.03 |

hpi, hours post-infection; WBC, white blood cells; RBC, red blood cells; Hb, hemoglobin; Hct; hematocrit; MCV, mean corpuscular volume; MCH, mean corpuscular hemoglobin; MCHC, mean corpuscular hemoglobin concentration; PLT, platelets; LYM, lymphocytes; NEUT; neutrophils, MONO; monocytes, EO, eosinophils; BASO, basophils. Data are presented as the mean ± SEM. *P* values were determined using 2-tailed unpaired Student’s *t*-test**.** **P*<0.05, ***P*<0.01
