# Supplementary material for: The zinc transporter Slc30a1 (ZnT1) in macrophages plays a protective role against attenuated Salmonella
Source: eLife. 2024 Oct 30;13:e89509. doi: 10.7554/eLife.89509 (PMC11524588; doi:10.7554/eLife.89509)
Supplement: Supplementary file 4. [file elife-89509-supp4.docx]

**Supplementary file 4.** List of primer pairs used for RT-qPCR analysis.

| Gene name | Forward primer (5’→3’) | Reverse primer (5’→3’) | Product size (bp) |
| --- | --- | --- | --- |
| *Slc30a1* | CCAACACCAGCAATTCCAAC | CTTGTACATCCACTGGGTCATC | 84 |
| *Mt1* | CCTGCAAGAACTGCAAGTG | TGGGCACATTTGGAGCA | 79 |
| *Tnfα* | CCCTCACACTCAGATCATCTTCT | GCTACGACGTGGGCTACAG | 61 |
| *Il1β* | CCCAACTGGTACATCAGCAC | TCTGCTCATTCACGAAAAGG | 180 |
| *Il6* | CGGAGAGGAGACTTCACAGA | CCAGTTTGGTAGCATCCATC | 218 |
| *Arg1* | CTCCAAGCCAAAGTCCTTAGAG | AGGAGCTGTCATTAGGGACATC | 185 |
| *Nos2* | TCACCTTCGAGGGCAGCCGA | TCCGTGGCAAAGCGAGCCAG | 286 |
| *Ccl2* | GGCTCAGCCAGATGCAGTT | TCTCCAGCCTACTCATTGGGA | 83 |
| *Cxcl1* | ACTGCACCCAAACCGAAGTC | TGGGGACACCTTTTAGCATCTT | 114 |
| *Cxcl10* | GACGGTCCGCTGCAACTG | GCTTCCCTATGGCCCTCATT | 65 |
| *p65* | TCCTGTTCGAGTCTCCATGCAG | GGTCTCATAGGTCCTTTTGCGC | 133 |
| *MyD88* | CACTCGCAGTTTGTTGGAT | CCACCTGTAAAGGCTTCTC | 185 |
| *Ikkα* | GCAGACCGTGAACATCCTCT | TCCAGGACAGTGAACGAGTG | 202 |
| *Ikkβ* | AGGCGACACGTGAACAGAT | CTAAGAGCGGATGCGATG | 298 |
